# Mucosal unadjuvanted booster vaccines elicit local IgA responses by conversion of pre-existing immunity in mice

**DOI:** 10.1038/s41590-025-02156-0

**Published:** 2025-05-13

**Authors:** Dong-il Kwon, Tianyang Mao, Benjamin Israelow, Keyla Santos Guedes de Sá, Huiping Dong, Akiko Iwasaki

**Affiliations:** 1 Department of Immunobiology, Yale School of Medicine, New Haven, CT USA; 2 Center for Infection and Immunity, Yale School of Medicine, New Haven, CT USA; 3 Howard Hughes Medical Institute, Chevy Chase, MD USA; 4 Section of Infectious Diseases, Department of Medicine, Yale School of Medicine, New Haven, CT USA; 5 Present Address: Broad Institute of MIT and Harvard, Cambridge, MA USA

**Keywords:** Adaptive immunity, Vaccines

## Abstract

Mucosal delivery of vaccine boosters induces robust local protective immune responses even without any adjuvants. Yet, the mechanisms by which antigen alone induces mucosal immunity in the respiratory tract remain unclear. Here we show that an intranasal booster with an unadjuvanted recombinant SARS-CoV-2 spike protein, after intramuscular immunization with 1 μg of mRNA–LNP vaccine encoding the full-length SARS-CoV-2 spike protein (Pfizer/BioNTech BNT162b2), elicits protective mucosal immunity by retooling the lymph node-resident immune cells. On intranasal boosting, peripheral lymph node-primed B cells rapidly migrated to the lung through CXCR3–CXCL9 and CXCR3–CXCL10 signaling and differentiated into antigen-specific IgA-secreting plasma cells. Memory CD4^+^ T cells in the lung served as a natural adjuvant for developing mucosal IgA by inducing the expression of chemokines CXCL9 and CXCL10 for memory B cell recruitment. Furthermore, CD40 and TGFβ signaling had important roles in mucosal IgA development. Repeated mucosal boosting with an unadjuvanted protein amplified anamnestic IgA responses in both the upper and the lower respiratory tracts. These findings help explain why nasal boosters do not require an adjuvant to induce robust mucosal immunity at the respiratory mucosa and can be used to design safe and effective vaccines against respiratory pathogens.

## Abstract (web summary)

Iwasaki and colleagues show that an intranasal booster with an unadjuvanted recombinant SARS-CoV-2 spike protein, after intramuscular immunization with an mRNA vaccine encoding the full-length SARS-CoV-2 spike protein, elicits protective mucosal immunity by retooling the lymph node-resident memory B cells in a manner dependent on the CD4^+^ T cells in the respiratory mucosa.

## Main

Induction of mucosal virus-specific IgA response is key to preventing SARS-CoV-2 infection and transmission^1–3^. Parenteral vaccination alone fails to induce strong IgA responses in the respiratory mucosa^4–6^, potentially leaving the host susceptible to transmission and breakthrough infection. However, parenteral priming, followed by nasal boosting, has been reported to robustly elicit mucosal immune responses that reduce SARS-CoV-2 infection and transmission, including IgA and tissue-resident memory lymphocytes^1,4,7–12^, even in the absence of any adjuvants^7,8^. Unadjuvanted nasal boosters are expected to be safer than adjuvanted vaccines because of a lack of innate immune stimuli that could induce inflammation. An inactivated influenza vaccine formulation with *Escherichia coli*-derived, heat-liable enterotoxin adjuvant given intranasally (i.n.) was linked to an increased risk of developing Bell’s palsy, caused by inflammation and swelling of the facial nerve^13^. Despite these observations, the mechanisms that drive antigen-specific IgA development at mucosal sites on boosting i.n. with an unadjuvanted protein remain unclear.

In the present study, we showed that systemic immunization with an mRNA–lipid nanoparticle (LNP) vaccine followed by an unadjuvanted nasal booster leveraged pre-existing immunity to elicit mucosal recall IgA responses. During parenteral priming, lymph node (LN)-resident memory B cells were established and later recruited to the lung and rapidly differentiated into antigen-specific IgA^+^ plasma cells on mucosal boosting. With an unadjuvanted nasal booster, CD4^+^ T cells functioned as natural adjuvants to recruit memory B cells and generate IgA-secreting plasma cells in the respiratory mucosa. These findings indicate how an unadjuvanted protein nasal booster transformed systemic immunity into localized mucosal recall responses and provided mechanistic insights for developing safe and effective mucosal vaccines against respiratory pathogens.

## Results

### Unadjuvanted nasal boosters elicit mucosal IgA recall responses

**Fig. 1:**
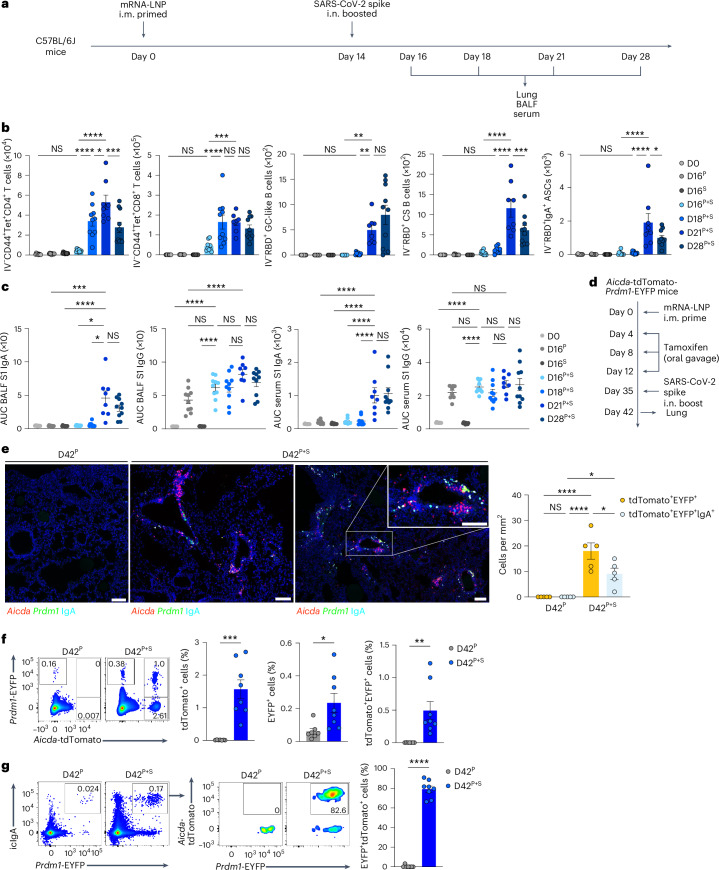
Unadjuvanted nasal booster elicits generation of antigen-specific IgA^+^ plasma cells at the respiratory mucosa. **a**, Schematic of the experimental setup showing C57BL/6J mice immunized i.m. with mRNA–LNPs encoding the full-length SARS-CoV-2 spike protein at day 0, i.n. boosted with an unadjuvanted SARS-CoV-2 spike protein at day 14, followed by lung, BALF and serum collection at days 16, 18, 21 and 28 post-priming i.m. **b**, Number of extravascular CD44^+^Tet^+^CD4^+^ T cells, CD44^+^Tet^+^CD8^+^ T cells, RBD^+^CD38^−^GL7^+^ GC-like B cells, RBD^+^CD38^+^GL7^−^IgM^−^IgD^−^ class-switched (CS) B cells and RBD^+^IgA^+^ ASCs in the lungs of naive C57BL/6J mice (day 0 (D0), *n* = 9); intramuscular prime-only mice (D16^P^, *n* = 10) and intranasal booster-only mice (D16^S^, *n* = 10) at day 16 post-priming i.m. and P + S mice at day 16 (D16^P+S^, *n* = 10), day 18 (D18^P+6^, *n* = 10), day 21 (D21^P+S^, *n* = 8) and day 28 (D128^P^, *n* = 10) post-priming i.m., analyzed by flow cytometry as in **a**. **c**, ELISA measurements of SARS-CoV-2 spike S1 subunit-specific IgA and IgG in the BALF and serum from mice as in **b**. AUC, area under the curve. **d**, Schematic of experiment showing *Aicda*-tdTomato-*Prdm1*-EYFP mice immunized i.m. with mRNA–LNPs at day 0, treated with 10 mg of tamoxifen via oral gavage at days 4, 8 and 12, boosted i.n. with a spike protein at day 35, followed by lung collection at day 42 post-priming. **e**, Immunofluorescence of IgA staining (left) and quantification (right) of colocalization of tdTomato^+^EYFP^+^ and tdTomato^+^EYFP^+^IgA^+^ cells in the lungs of intramuscular prime-only (D42^P^, *n* = 5) or P + S (D42^P+S^, *n* = 5) *Aicda*-tdTomato-*Prdm1*-EYFP mice at day 42 post-priming i.m. (day 7 post-boosting i.n.) as in **d**. Scale bar, 100 μm. **f**, Representative flow cytometry plots and frequency of EYFP^+^, tdTomato^+^ and tdTomato^+^EYFP^+^ cells in the lungs of intramuscular prime-only (D42^P^, *n* = 7) and P + S (D42^P+S^, *n* = 8) *Aicda*-tdTomato-*Prdm1*-EYFP mice analyzed by flow cytometry at day 42 post-priming i.m. as in **d**. **g**, Representative flow cytometry plots and frequency of tdTomato^+^ cells among EYFP^+^IgA^+^ cells in the lungs of mice as in **f** (mean ± s.e.m.). Statistical significance was calculated using one-way ANOVA (**b**,**c**,**e**) and unpaired Student’s *t-*test (**f**,**g**). Tukey’s multiple comparisons (**b**,**c**,**e**) were performed: *P < 0.05, **P < 0.01, ***P < 0.001, ****P < 0.0001. NS, not significant. Data were pooled from three (**b**,**c**) independent experiments or represented (**e**–**g**) two independent experiments. Values indicated as zero represent the absence of detectable cells (**b**,**e–g**). Source data

**Extended Data Fig. 1:**
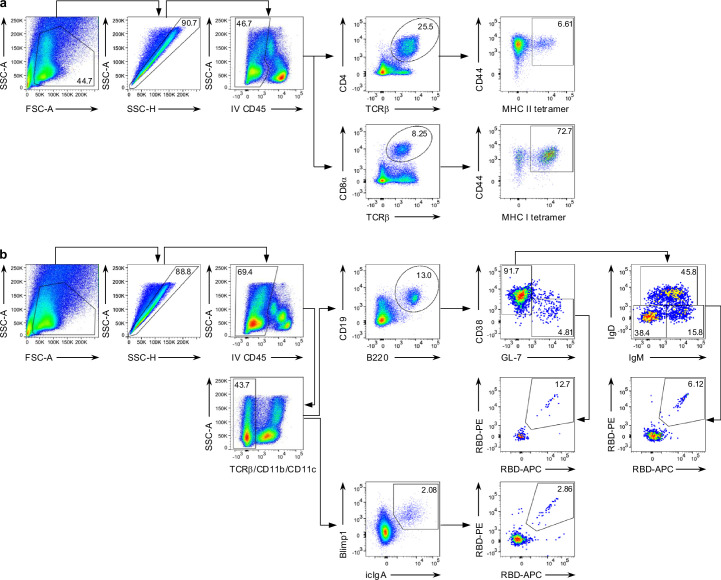
Gating strategies for extravascular antigen-specific T and B cell analysis in the lung. **a,b**, C57BL/6J mice were immunized i.m. with mRNA-LNPs encoding the full-length SARS-CoV-2 spike protein (Pfizer/BioNTech BNT162b2) at day 0, followed by intranasal boosting with an unadjuvanted recombinant SARS-CoV-2 spike protein at day 14 post-priming as in Fig. 1a. Gating strategies of extravascular CD44^+^Tet^+^CD4^+^ T cells (upper row) and CD44^+^Tet^+^CD8^+^ T cells (lower row) (**a**) as well as RBD^+^CD38^-^GL7^+^ GC-like B cells, RBD^+^CD38^+^GL7^-^IgM^-^IgD^-^ class-switched (CS) B cells (upper and middle row), and RBD^+^IgA^+^ antibody-secreting cells (ASCs) (lower row) (**b**) in the lung of i.m. prime+i.n. booster (P + S) mice at day 7 post i.n. boosting.

We first assessed the time course of accumulation of pulmonary antigen-specific T cells and B cells during the ‘prime and spike’ (hereafter P + S) vaccine approach^7^, in which C57BL/6J mice were injected intramuscularly (i.m.) with a 1 μg mRNA–LNP vaccine encoding the full-length SARS-CoV-2 spike protein (Pfizer/BioNTech BNT162b2) at day 0, followed by boosting i.n. with an unadjuvanted 1 μg SARS-CoV-2 spike protein at day 14 (Fig. 1a). CD45 labeling intravenously (i.v.) and spike-specific major histocompatibility complex class I (MHC-I) and MHC-II tetramers indicated a notable increase in the number of spike-specific, tissue-resident CD4^+^ and CD8^+^ T cells in the lungs at day 18 after primary vaccination, with a peak at day 21 post-priming i.m. (Fig. 1b and Extended Data Fig. 1a). Next, we identified antigen-specific, tissue-resident B cells and IgA^+^ antibody-secreting cells (ASCs) using SARS-CoV-2 receptor-binding domain (RBD)-specific tetramers and intracellular staining with antibodies specific for IgA and BLIMP1, a transcription factor essential for plasma cell generation^14^. At day 7 after boosting i.n., we detected a substantial increase in tissue-resident, RBD-specific CD38^−^GL7^+^ germinal center (GC)-like and CD38^+^GL7^−^IgM^−^IgD^−^ class-switched B cells, and icIgA^+^BLIMP1^+^ ASCs in the lungs (Fig. 1b and Extended Data Fig. 1b). Notably, only mice that received the booster i.n. showed an increase in SARS-CoV-2 S1 subunit-specific IgA amount in the bronchoalveolar lavage fluid (BALF) and serum (Fig. 1c). Although priming with mRNA–LNP vaccine alone elicited S1-specific IgG in the serum, BALF S1-specific IgG levels increased at day 21 post-priming i.m. (Fig. 1c). Thus, unadjuvanted spike protein delivery i.n. rapidly triggered strong antigen-specific CD4^+^ and CD8^+^ T cell and IgA responses in the lung.

**Extended Data Fig. 2:**
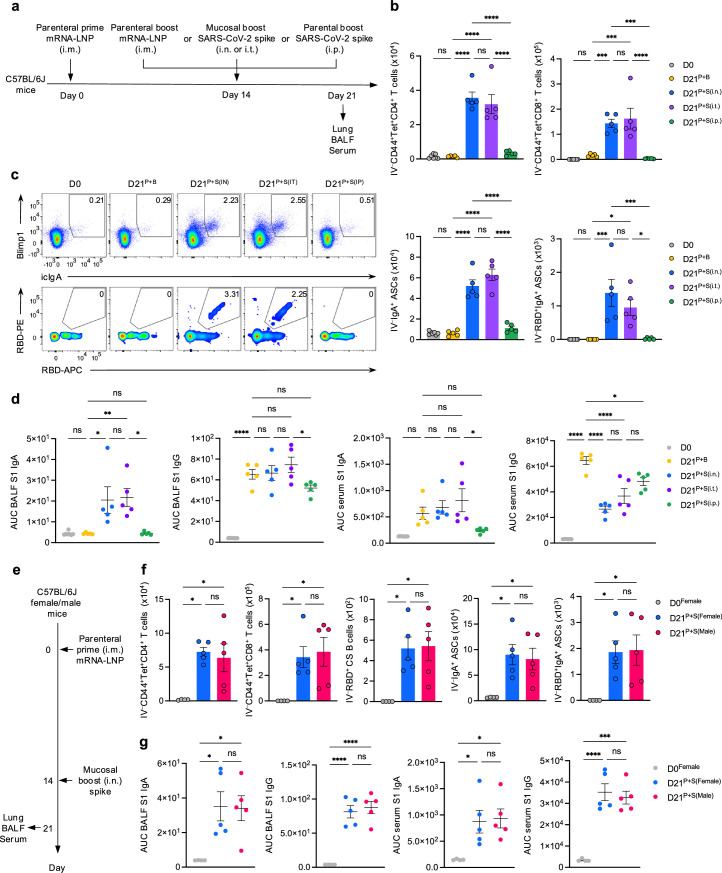
Direct antigen delivery into the lung is essential for mucosal recall responses, which are comparable between male and female C57BL/6 mice. **a**, Schematic of the experimental setup showing i.m. primed C57BL/6 mice with mRNA-LNPs (day 0) were i.m. boosted with mRNA-LNPs or i.n. or i.t. or i.p. boosted with an unadjuvanted SARS-CoV-2 spike protein at day 14 post-priming, followed by lung, BALF, serum collection at day 7 post-boosting. **b**, Number of extravascular IV^–^CD44^+^Tet^+^CD4^+^ T cells and IV^–^CD44^+^Tet^+^CD8^+^ T cells of naïve C57BL/6J mice (D0, n = 7); i.m. primed+i.m. boosted mice (D21^P+B^, n = 5); i.m. primed+i.n. boosted mice (D21^P+S(i.n.)^, n = 5); i.m. primed+i.t. boosted mice (D21^P+S(i.t.)^, n = 5); i.m. primed+i.p. boosted mice (D21^P+S(i.p.)^, n = 5) at day 21 post-priming analyzed by flow cytometry as in **a. c**, Representative flow cytometry plots and quantification of the number of total and RBD-specific IgA^+^ ASCs in the lung of mice as in **b**. **d**, ELISA measurements of SARS-CoV-2 spike protein S1 subunit-specific IgA and IgG in the BALF and serum from mice as in **b. e**, Schematic of the experimental setup showing female and male C57BL/6J mice were i.m. primed with mRNA-LNPs at day 0, i.n. boosted with an unadjuvanted spike protein at day 14, followed by lung, BALF, and serum collection at day 21 post-priming. **f**, Number of extravascular CD44^+^Tet^+^CD4^+^ T cells, CD44^+^Tet^+^CD8^+^ T cells, RBD^+^CD38^+^GL7^-^IgM^-^IgD^-^ CS B cells, total IgA^+^ ASCs, and RBD^+^IgA^+^ ASCs in the lung of naïve female C57BL/6 mice (D0^Female^, n = 4); P + S female (D21^P+S(Female)^, n = 5) and male (D21^P+S(Male)^, n = 5) mice at day 21 post-priming analyzed by flow cytometry as in **e**. **g**, ELISA measurements of SARS-CoV-2 S1-specific IgA and IgG in the BALF and serum from mice as in **f**. Mean ± SEM. Statistical significance was calculated by one-way ANOVA (**b,c,d,f,g**). Tukey’s multiple comparisons were performed; *P < 0.05, **P < 0.01, ***P < 0.001, ****P < 0.0001. Data were pooled from two (**b,c,d,f,g**) independent experiments and representative (**f,g**) of an experiment. Values shown as zero represent the absence of detectable cells (**b,c,f**). Source data

To determine whether direct delivery of unadjuvanted nasal boosters into the lungs was required for eliciting mucosal recall responses, we compared antigen-specific T and B cell immunity in C57BL/6J mice primed with 1μg mRNA–LNPs at day 0, followed by boosting at day 14 with either 1 μg of mRNA–LNPs i.m. or 1 μg of SARS-CoV-2 spike protein i.n., intratracheally (i.t.), or intraperitoneally (i.p.) (Extended Data Fig. 2a). Boosting i.t. with an unadjuvanted SARS-CoV-2 spike protein substantially increased the number of spike-specific CD4^+^ and CD8^+^ T cells, as well as RBD-specific IgA^+^ ASCs in the lungs compared with boosting i.m. with mRNA–LNPs and i.p. with spike protein (Extended Data Fig. 2b). Boosting i.m. with mRNA–LNPs and systemic boosting i.p. with SARS-CoV-2 spike protein did not induce RBD-specific IgA^+^ ASCs in the lung (Extended Data Fig. 2c). Only mice boosted i.n. or i.t. showed an increase in S1-specific IgA levels in BALF, whereas serum S1-specific IgG levels were higher in mice boosted i.m. with mRNA–LNPs at day 14 post-priming i.m. (Extended Data Fig. 2d). Thus, antigen delivery directly into the respiratory mucosa was necessary to generate robust local CD4^+^ and CD8^+^ T cell and IgA responses. The numbers of spike-specific CD4^+^ and CD8^+^ T cells, RBD-specific IgA^+^ ASCs and S1-specific IgA and IgG levels in BALF and serum were comparable between female and male mice boosted i.n. with spike protein at day 14 post-priming i.m. (Extended Data Fig. 2e–g), indicating that an unadjuvanted intranasal protein booster induced robust immune responses in respiratory mucosa independent of sex.

Next, we investigated the localization of IgA^+^ plasma cells and memory B cells in *Aicda*-ERT2-Cre-*Rosa26*-tdTomato *Prdm1*-enhanced yellow fluorescent protein (EYFP) mice treated with tamoxifen at days 4, 8 and 12 after immunization i.m. with mRNA–LNPs, followed by boosting i.n. with SARS-CoV-2 spike protein at day 35 (Fig. 1d), an experimental setup in which activated B cells are permanently tdTomato^+^ and primed B cell-derived plasma cells are tdTomato^+^EYFP^+^. Although not all activated B cells are fate mapped in the *Aicda*-ERT2-Cre mice^15^, we observed predominantly tdTomato^+^ primed B cell (red) clusters, along with a mixture of EYFP^+^ plasma cells (green), tdTomato^+^EYFP^+^ primed B cell-derived plasma cells (yellow) and tdTomato^+^EYFP^+^IgA^+^ plasma cells (white) derived from B cells primed i.m. near the blood vessels and airways in the lung parenchyma at day 42 post-priming i.m. (Fig. 1e). All these B cell populations were absent in intramuscular prime-only mice (Fig. 1e). Flow cytometry analysis of whole lung tissue also indicated a substantial increase in frequency of tdTomato^+^, EYFP^+^ and tdTomato^+^EYFP^+^ cells in the lungs of i.n. boosted compared with intramuscular prime-only *Aicda*-ERT2-Cre-*Rosa26*-tdTomato *Prdm1*-EYFP mice at day 42 post-priming i.m. (Fig. 1f). More than 80% of IgA^+^ plasma cells in the mice boosted i.n. were tdTomato^+^ (Fig. 1g), suggesting that IgA-producing plasma cells mainly originated from activated B cells formed during priming i.m. Thus, boosting i.n. with an unadjuvanted spike protein induced IgA^+^ plasma cell development in the respiratory mucosa.

### Mucosal IgA responses originate from circulating memory cells

**Fig. 2:**
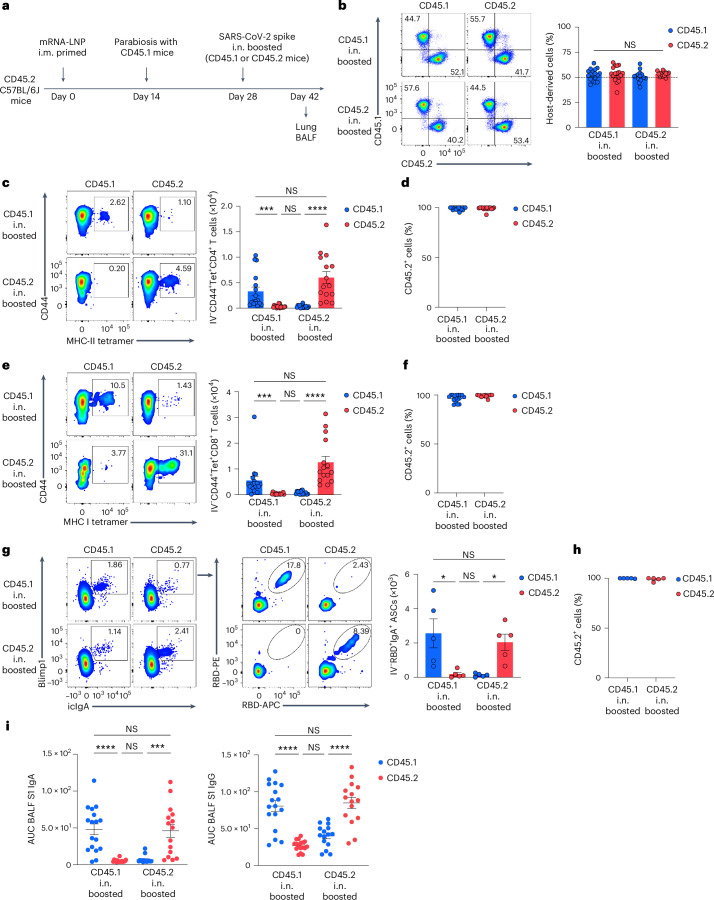
Circulating memory cells are the source for mucosal recall responses. **a**, Schematic of the experimental design showing CD45.2 C57BL/6J mice primed i.m. with mRNA–LNPs at day 0 surgically paired with CD45.1 naive mice at day 14, followed by an intranasal boost of either the CD45.1 naive or the CD45.2 primed mice of the parabiotic pair with SARS-CoV-2 spike protein at day 14 after surgery, and analysis of lungs and BALF at day 14 after booster i.n. **b**, Representative flow cytometry plots and frequency of host-derived cells among intravenous CD45^+^ cells in the lungs of a parabiosis pair boosted i.n. in either CD45.1 (*n* = 17) or CD45.2 (*n* = 15) mice analyzed by flow cytometry as in **a**. **c**–**f**, Representative flow cytometry plots (left) and number (right) of extravascular IV^−^CD44^+^Tet^+^CD4^+^ T cells (**c**) and IV^−^CD44^+^Tet^+^CD8^+^ T cells (**e**) and frequency of CD45.2^+^ cells among extravascular IV^−^CD44^+^Tet^+^CD4^+^ T cells (**d**) and IV^−^CD44^+^Tet^+^CD8^+^ T cells (**f**) in the lungs of CD45.1 and CD45.2 mice of a parabiosis pair as in **b**. **g**, Representative flow cytometry plots and extravascular RBD^+^IgA^+^ ASC numbers in the lungs of CD45.1 (*n* = 5) and CD45.2 (*n* = 5) mice of a parabiosis pair as in **a**. **h**, Frequency of CD45.2^+^ cells among extravascular IV^−^RBD^+^IgA^+^ ASCs in the lungs of CD45.1 and CD45.2 mice of a parabiosis pair as in **g**. **i**, ELISA measurements of SARS-CoV-2 S1-specific IgA and IgG in the BALF of CD45.1 and CD45.2 mice of a parabiosis pair as in **b**. Data are the mean ± s.e.m. The statistical significance was calculated using one-way ANOVA (**b**,**i**) or nonparametric Kruskal–Wallis test (**c**,**e**,**g**). Tukey’s multiple comparisons (**b**,**i**) and Dunn’s multiple comparisons (**c**,**e**,**g**) were performed: *P < 0.05, **P < 0.01, ***P < 0.001, ****P < 0.0001. Data were pooled from four (**b–f**) independent experiments or represent (**g**,**h**) an experiment. Values shown as zero indicate the absence of detectable cells (**c**,**e**,**g**). Source data

To identify the source of antigen-specific mucosal responses, CD45.2 mice primed i.m. with mRNA–LNPs at day 0 were surgically paired with CD45.1 naive mice at day 14 to establish parabiotic pairs in which the circulatory systems are known to equilibrate within 2 weeks^16^. At week 2 post-surgery, either the CD45.1 naive or the CD45.2 i.m. primed mice of the parabiosis pair received an unadjuvanted SARS-CoV-2 spike protein i.n. (Fig. 2a). At day 14 post-boosting i.n., the frequency of host-derived cells among intravenously (i.v.) CD45-labeled cells in the lung of each parabiont was comparable (Fig. 2b), indicating that the circulatory immune cells had reached an equilibrium. The number of spike-specific CD4^+^ and CD8^+^ T cells was markedly higher in the lung of i.n. boosted parabiont compared with the non-i.n. boosted partner parabiont (Fig. 2c,e). Notably, >90% of the spike-specific CD4^+^ and CD8^+^ T cells originated from CD45.2^+^ T cells derived from the i.m. primed parabiont (Fig. 2d,f), suggesting that mucosal recall responses were mostly derived from circulating memory T cells rather than naive T cells after boosting i.n. RBD-specific IgA^+^ ASCs were predominantly present in the lung of the i.n. boosted parabiont and most of them were CD45.2^+^ (Fig. 2g,h), suggesting that they originated from the i.m. primed parabiont. BALF S1-specific IgA was markedly elevated in the i.n. boosted parabionts, but undetectable in the non-i.n. boosted parabiont (Fig. 2i), indicating local production and secretion of IgA. S1-specific IgG levels were higher in the BALF of the i.n. boosted parabionts compared with the non-i.n. boosted parabiont (in which the BALF S1 IgG was still highly detectable) (Fig. 2i), suggesting that BALF S1-specific IgG was primarily derived from systemic sources after boosting i.n. Thus, circulating immune cells were sufficient to elicit antigen-specific antibody responses in the lung after boosting i.n. and local mucosal T cell and IgA responses that developed after this nasal booster with an unadjuvanted SARS-CoV-2 spike protein originated from those primed during the intramuscular mRNA–LNP vaccination.

### Lymph node memory B cells are the main source of mucosal IgA responses

**Fig. 3:**
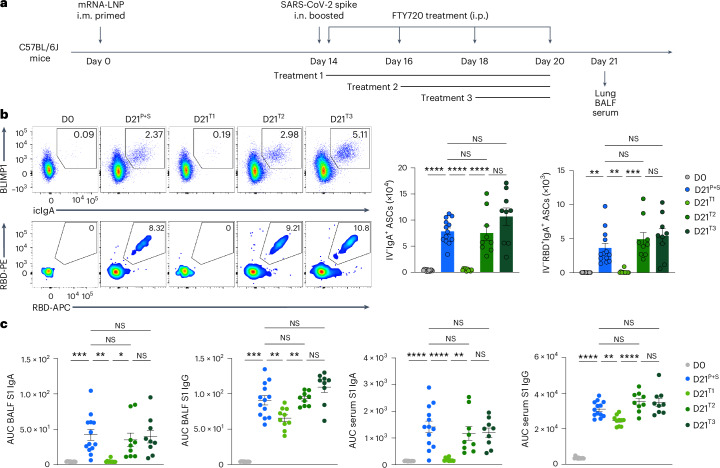
Lymph node egress is necessary to generate anamnestic mucosal IgA responses. **a**, Schematic of the experimental setup showing C57BL/6J mice i.m. primed with mRNA–LNPs at day 0, i.n. boosted with an unadjuvanted SARS-CoV-2 spike protein at day 14 post-priming, followed by treatment i.p. with PBS or FTY720 every other day, starting at day 14 (treatment 1), 16 (treatment 2) or 18 (treatment 3) after intramuscular priming, and lung, BALF and serum collection at day 21 after an intramuscular boost. **b**, Representative flow cytometry plots and number of extravascular total and RBD-specific IgA^+^ ASCs in the lungs of naive C57BL/6J mice (D0, *n* = 12); P + S mice treated with PBS (D21^P+S^, *n* = 13) or P + S mice treated with FTY720 at day 14 (D21^T1^, *n* = 10); day 16 (D21^T2^, *n* = 9); P + S day 18 (D21^T3^, *n* = 9) analyzed by flow cytometry at day 21 after intranasal priming as in **a**. **c**, ELISA measurements of SARS-CoV-2 S1-specific IgA and IgG in the BALF and serum from mice as in **b**. Data are the mean ± s.e.m. The statistical significance was calculated using one-way ANOVA (**b**,**c**). Tukey’s multiple comparisons were performed: *P < 0.05, **P < 0.01, ***P < 0.001, ****P < 0.0001. Data were pooled from three independent experiments (**b**,**c**). Values indicated as zero show the absence of detectable cells (**b**). Source data

**Extended Data Fig. 3:**
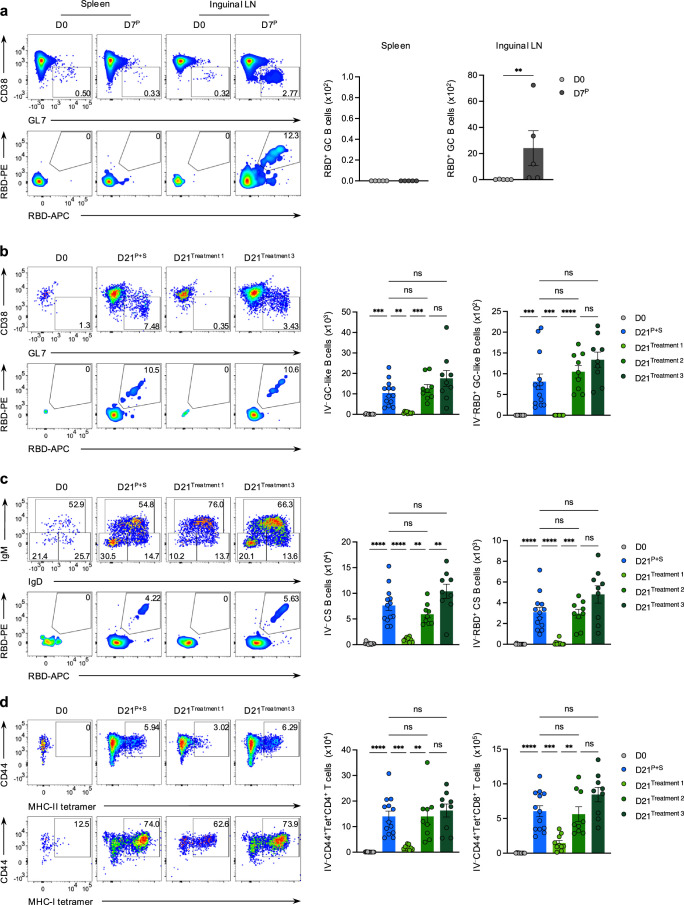
Mucosal recall T and B cell response requires LN egress upon mucosal boosting with an unadjuvanted protein. **a**, Representative flow cytometry plots and the number of RBD-specific CD38^-^GL7^+^ GC B cells in the spleen and inguinal lymph node of naïve C57BL/6J mice (D0, n = 5) and mRNA-LNP i.m. primed mice (D7^P^, n = 5) at day 7 post-priming analyzed by flow cytometry. **b-d**, Representative flow cytometry plots and quantification of the number of total and RBD-specific IV^–^CD38^-^GL7^+^ GC-like B cells (**b**), IV^–^CD38^+^GL7^-^IgM^-^IgD^-^ CS B cells (**c**), IV^–^CD44^+^Tet^+^CD4^+^ T cells and IV^–^CD44^+^Tet^+^CD8^+^ T cells (**d**) in the lung of naïve C57BL/6J mice (D0, n = 12); P + S mice treated with PBS (D21^P+S^, n = 13) or P + S mice treated with FTY720 at day 14 (D21^T1^, n = 10); day 16 (D21^T2^, n = 9); P + S day 18 (D21^T3^, n = 9) analyzed by flow cytometry at day 21 post-priming as in Fig. 3a. Mean ± SEM. Statistical significance was calculated by unpaired t-test (**a**) and one-way ANOVA (**b-d**). Tukey’s multiple comparisons were performed; *P < 0.05, **P < 0.01, ***P < 0.001, ****P < 0.0001. Data were pooled from two (**b-d**) independent experiments and representative (**a**) of an experiment. Values indicated as zero represent the absence of detectable cells (**a-d**). Source data

Intramuscular vaccination with mRNA–LNPs induces a robust GC response in the draining lymph nodes in both mice and humans within 1–3 weeks^17–20^. Consistently, the mRNA–LNP vaccination i.m. notably induced antigen-specific GC B cell responses in the inguinal lymph nodes, but not in the spleen of C57BL/6J mice at day 7 after immunization i.m. (Extended Data Fig. 3a). As the origin of memory B cells that seed the antigen-specific IgA^+^ B cells in the lung mucosal tissues remains unknown, we investigated whether lymph nodes contained B cells that could be induced to secrete IgA on boosting i.n. C57BL/6J mice primed i.m. with mRNA–LNPs (day 0) were treated with phosphate-buffered saline (PBS) or the S1PR1 agonist FTY720, which inhibits lymphocyte egress from the secondary lymphoid tissues, every other day for a week starting at days 0, 2 or 4 post-boosting i.n. (day 14) with an unadjuvanted SARS-CoV-2 spike protein (Fig. 3a). Lung RBD-specific IgA^+^ ASCs, CD38^−^GL7^+^ GC-like B cells and CD38^+^GL7^−^IgM^−^IgD^−^ class-switched B cells disappeared almost entirely in the i.n. boosted mice treated with FTY720 at day 0 after an intranasal booster, but were detected in i.n. boosted mice treated with FTY720 at days 2 and 4 with similar levels to i.n. boosted mice not treated with FTY720 (Fig. 3b and Extended Data Fig. 3b,c). BALF S1-specific IgA levels were almost undetectable in i.n. boosted, day 0, FTY720-treated mice, but remained unaffected in i.n. boosted, day 2 and 4 FTY720-treated mice (Fig. 3c). S1-specific BALF IgG and serum IgA levels were substantially reduced, whereas serum S1-specific IgG was minimally affected in i.n. boosted, day 0, FTY720-treated mice compared with i.n. boosted, PBS-treated mice (Fig. 3c). These observations indicated that IgA^+^ B cells in the lung originated in the lymph nodes and exited within 2 d of boosting i.n. In addition, the number of i.n. boosted, spike-specific, lung CD4^+^ and CD8^+^ T cells were markedly reduced in day 0, but not day 2 or 4, FTY720-treated mice, compared with PBS-treated mice (Extended Data Fig. 3d), suggesting that lymph node egress was required for the induction of mucosal T cell and antibody responses.

**Extended Data Fig. 4:**
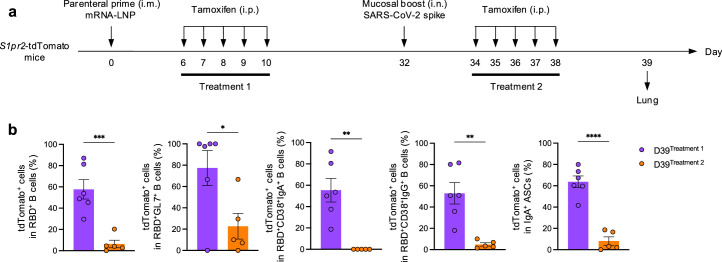
Mucosal B cell response mainly originates from GC-derived cells formed during primary immunization. **a**, Schematic of experimental showing S1pr2-tdTomato mice were i.m. immunized with mRNA-LNPs at day 0, i.n. boosted with an unadjuvanted SARS-CoV-2 spike protein at day 32, treated i.p. with 2 mg of 4-hydroxytamoxifen at days 6,7,8,9, and 10 (treatment 1) or at days 34, 35, 36, 37, and 38 (treatment 2) post intramuscular priming, followed by lung collection at day 39 post-priming. **b**, Frequency of tdTomato^+^ cells among RBD^+^ B cells, RBD^+^GL7^+^ B cells, RBD^+^CD38^+^IgA^+^ B cells, RBD^+^CD38^+^IgG^+^ B cells, and IgA^+^ ASCs in the lung of tamoxifen treated S1pr2-tdTomato mice post intramuscular priming (D39^T1^, n = 6) or post intranasal boosting (D39^T2^, n = 5) analyzed flow cytometry at day 39 post-priming as in **a**. Mean +/− SEM. Statistical significance was calculated by an unpaired t-test. *P < 0.05, **P < 0.01, ***P < 0.001, ****P < 0.0001. Data were pooled from two (**b**) independent experiments. Values shown as zero indicate the absence of detectable cells (**b**). Source data

To examine whether mucosal B cells originated from primary GC-derived cells, *S1pr2*-ERT2-Cre-*Rosa26*-tdTomato mice, which allow fate mapping of tdTomato^+^ GC B cells after tamoxifen treatment, were i.m. primed with mRNA–LNPs at day 0 and i.n. boosted with an unadjuvanted SARS-CoV-2 spike protein at day 32 after intramuscular priming. Intramuscularly primed *S1pr2*-ERT2-Cre-*Rosa26*-tdTomato mice were i.p. treated with 4-hydroxytamoxifen at days 6, 7, 8, 9 and 10 after priming i.m. or at days 2, 3, 4, 5 and 6 after boosting i.n. (Extended Data Fig. 4a). At day 39 post-primary immunization, >90% of lung RBD-specific B cells and IgA^+^ ASCs were tdTomato^−^ in mice treated with tamoxifen after boosting i.n. (Extended Data Fig. 4b). By contrast, >60% of them were tdTomato^+^ in mice given tamoxifen after priming i.m. (Extended Data Fig. 4b), indicating that lung mucosa IgA recall responses were derived from GC B cells formed during primary immunization i.m., not post-boosting i.n. Thus, lymph node-resident memory B cells formed during primary vaccination have a critical role in mucosal recall IgA responses.

### CXCR3-dependent signaling recruits memory lymphocytes

**Extended Data Fig. 5:**
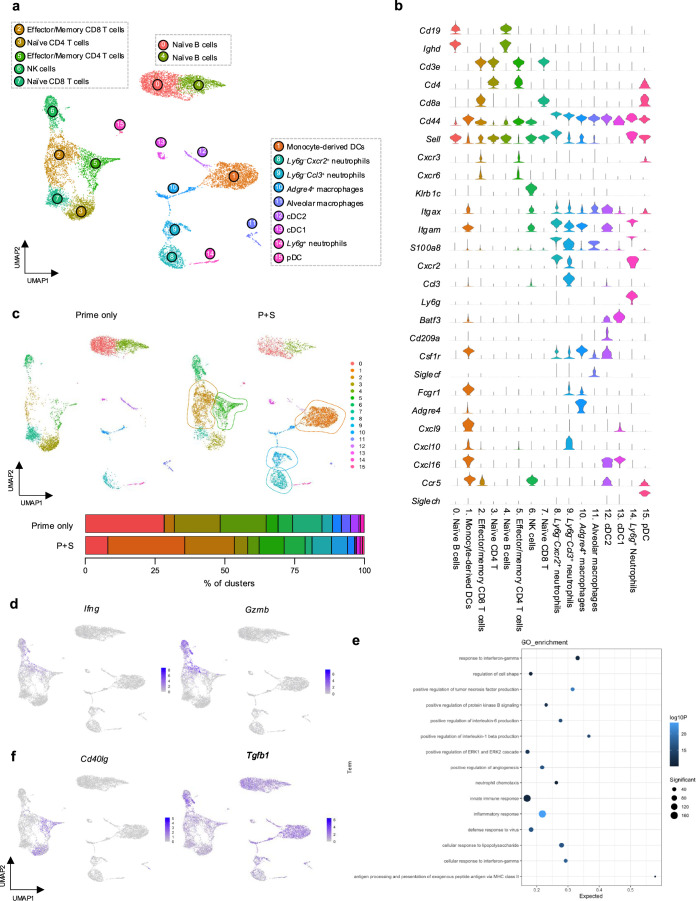
scRNA-seq analysis reveals early changes in the lung immune cell populations following mucosal boosting. **a**, Uniform manifold approximation and projection (UMAP) plot of extravascular CD45^+^ cells in the lung from i.m. primed only and P + S C57BL/6 mice at 2 days post intranasal boosting with an unadjuvanted spike protein. Each immune cell population was labeled with the indicated colors**. b**, Violin plots showing expression levels of indicated marker genes in each immune cell cluster as in **a**. **c**, UMAP plot and frequency of clusters from mice as in **a**. **d**, UMAP plots of *Ifng* and *Gzmb* expression. **e**, Gene ontology analysis using upregulated genes by intranasal booster compared to i.m. prime only. **f**, UMAP plots showing *Cd40lg* and *Tgfb1* expression.

To identify early changes in immune cell populations on boosting i.n., we performed single-cell RNA sequencing (scRNA-seq) of extravascular lung CD45^+^ cells from P + S C57BL/6J mice at day 2 after intranasal boosting. Among the 16 clusters identified, i.n. boosted mice had increased subsets of *Ifng*^+^*Gzmb*^+^ natural killer (NK) cells (cluster C6) and memory or activated *Cd44*^+^*Cxcr3*^+^*Cd4*^+^ and *Cd44*^+^*Cxcr3*^+^*Cd8*^+^ T cells (C3 and C5), as well as *Cxcl9*^+^*Cxcl10*^+^ monocyte-derived dendritic cells (moDCs, C1) and *Ly6g*^−^*Cxcr2*^+^ and *Ly6g*^−^*Ccl3*^+^ neutrophils (C8 and C9) compared with i.m. primed mice (Extended Data Fig. 5a–d), suggesting that the intranasal booster induced the recruitment of NK cells, memory T (T_M_) cells and innate immune cells to the lungs. Gene Ontology (GO) analysis showed that genes upregulated at day 2 after boosting i.n. compared with priming i.m. only were associated with inflammatory response, antigen processing and presentation and defense response to the virus (Extended Data Fig. 5e). Also, effector or memory CD4^+^ T cell clusters expressed *Cd40lg* and *Tgfb1*, which are essential for T cell-dependent, IgA class-switch recombination (CSR)^21–25^ (Extended Data Fig. 5f). Thus, an unadjuvanted spike intranasal booster shaped immune-stimulating microenvironmental niches to elicit anamnestic immunity in the lung.

**Fig. 4:**
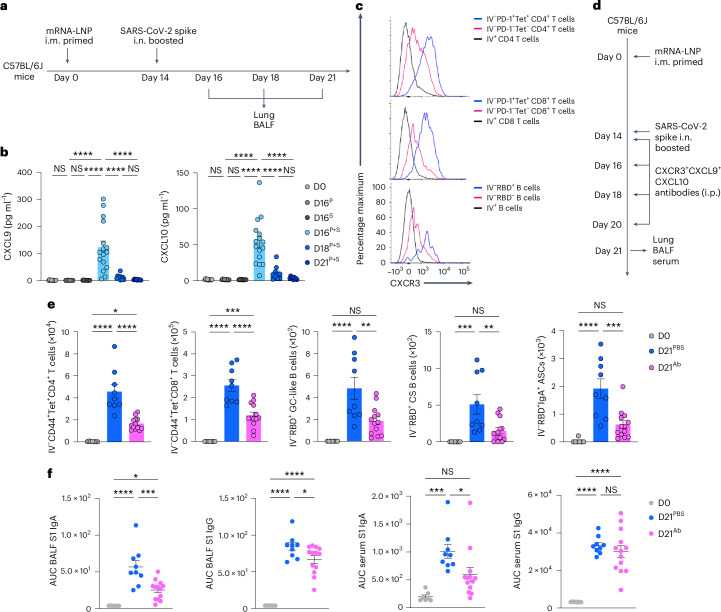
CXCR3–CXCL9 or CXCR3–CXCL10 signaling is crucial for memory T cell and B cell recruitment into the lung on mucosal boosting. **a**, Schematic of the experimental setup showing C57BL/6J mice immunized i.m. with mRNA–LNPs at day 0, i.n. boosted with an unadjuvanted SARS-CoV-2 spike protein at day 14, followed by lung and BALF collection at days 16, 18 and 21 after priming i.m. **b**, ELISA measurements of CXCL9 and CXCL10 concentrations in the BALF from naive C57BL/6J mice (D0, *n* = 10); intramuscular prime-only (D16^P^, *n* = 9) and intranasal booster-only (D16^S^, *n* = 10) mice at day 16; and P + S mice at day 16 (D16^P+S^, *n* = 15), day 18 (D18^P+S^, *n* = 10) and day 21 (D121^P+S^, *n* = 9) as in **a**. Detection limits, CXCL9 (0.23 pg ml^−1^) and CXCL10 (0.63 pg ml^−1^). **c**, Representative histogram of CXCR3 expression in extravascular IV^−^PD-1^+^Tet^+^ and IV^−^PD-1^−^Tet^−^ and circulating IV^+^CD4^+^ T and CD8^+^ T cells and extravascular IV^−^RBD^+^ and IV^−^RBD^−^ and circulating IV^+^ B cells in the lungs of P + S mice at day 21 post-priming. **d**, Schematic of the experimental design showing C57BL/6J mice immunized i.m. with mRNA–LNPs at day 0, i.n. boosted with an unadjuvanted SARS-CoV-2 spike protein at day 14, followed by being i.p. treated with PBS or CXCR3 + CXCL9 + CXCL10 neutralizing antibodies at days 14, 16, 18 and 20 after intramuscular priming and lungs, BALF and serum were analyzed at day 21 after priming i.m. **e**, Number of extravascular IV^−^CD44^+^Tet^+^CD4^+^ T cells, IV^−^CD44^+^Tet^+^CD8^+^ T cells, IV^−^RBD^+^CD38^−^GL7^+^ GC-like B cells, IV^−^RBD^+^CD38^+^GL7^−^IgM^−^IgD^−^ class-switched B cells and IV^−^RBD^+^IgA^+^ ASCs in the lung of naive mice (D0, *n* = 7) and P + S mice treated with PBS (D21^PBS^, *n* = 9) or CXCR3 + CXCL9 + CXCL10-neutralizing antibodies (D21^Ab^, *n* = 13) at day 21 after priming i.m. as in **d** analyzed by flow cytometry. **f**, ELISA measurements of SARS-CoV-2 spike S1-specific IgA and IgG in the BALF and serum from mice as in **e**. Data are the mean ± s.e.m. The statistical significance was calculated using one-way ANOVA (**b**,**e**,**f**). Tukey’s multiple comparisons were performed: *P < 0.05, **P < 0.01, ***P < 0.001, ****P < 0.0001. Data were pooled from three (**b**) or two independent (**e**,**f**) experiments or represent (**c**) two independent experiments. Values shown as zero indicate the absence of detectable cells (**b**,**e**). Source data

To address whether early changes in the lung mucosa innate cell populations induced recruitment of memory lymphocytes to the lung, we measured the production of chemokines in BALF of P + S C57BL/6 mice at days 2, 4 and 7 after boosting i.n. with SARS-CoV-2 spike protein by ELISA (Fig. 4a). Expression of CXCL9 and CXCL10, which are strong chemoattractants for T_M_ cells and memory B (B_M_) cells^26^, was markedly elevated at day 2 after boosting i.n. and rapidly decreased at day 4 after boosting i.n. (Fig. 4b). At day 7 after boosting i.n., lung Tet^+^PD-1^+^CD4^+^ T cells, Tet^+^PD-1^+^CD8^+^ T cells and RBD^+^ B cells had much higher expression of CXCR3 compared with lung tetramer^−^ or circulating i.v. CD45-labeled CD4^+^ T cells, CD8^+^ T cells and B cells analyzed by flow cytometry (Fig. 4c). To test the role of CXCR3–CXCL9 and CXC3–CXCL10 signaling in the recruitment of CD44^+^Tet^+^ T_M_ cells and RBD^+^CD38^+^IgD^−^ B_M_ cells to the lung, C57BL/6J mice i.m. primed with mRNA–LNPs were injected with blocking antibodies specific for CXCR3, CXCL9 and CXCL10 (to induce complete blockade of CXCR3–CXCL9-CXCL10 signaling) immediately after boosting i.n. with an unadjuvanted SARS-CoV-2 spike protein (Fig. 4d). We found a substantial reduction in CD44^+^Tet^+^CD4^+^ T cells, CD44^+^Tet^+^CD8^+^ T cells, RBD-specific CD38^−^GL7^+^ GC-like B cells, RBD-specific CD38^+^GL7^−^IgM^−^IgD^−^ class-switched B cells and RBD-specific IgA^+^ ASCs in the lungs (Fig. 4e), as well as S1-specific IgA in BALF and serum, but not serum S1-specific IgG (Fig. 4f) in mice treated with CXCR3 + CXCL9 + CXCL10-blocking antibodies compared with mice treated with PBS. These results indicated that CXCR3–CXCL9 and CXCR3–CXCL10 signaling recruited T_M_ cells and B_M_ cells required for the intranasal booster-mediated responses.

### Endotoxin-free nasal booster induces mucosal recall responses

**Extended Data Fig. 6:**
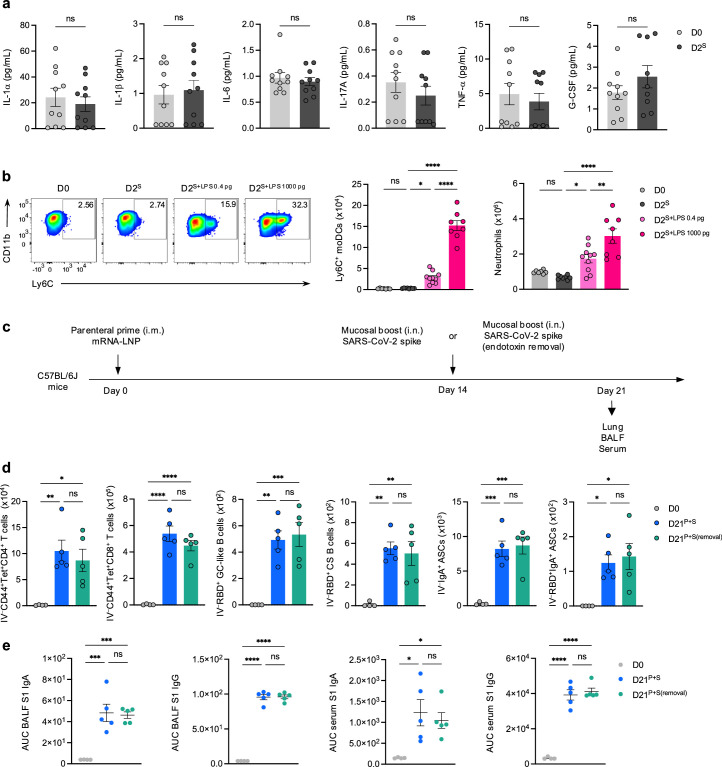
Mucosal recall responses induced by an unadjuvanted nasal booster are not due to endotoxin contamination. **a**, ELISA measurements of IL-1α, IL-1β, IL-6, IL-17A, TNF-α, and G-CSF concentrations in the BALF of naïve C57BL/6J mice (D0, n = 10) and unadjuvanted recombinant spike protein i.n. treated mice (D2^S^, n = 10) at day 2 post intranasal treatment. Detection limits, IL-1α (0.64 pg/mL), IL-1β (0.1 pg/mL), IL-6 (0.64 pg/mL), IL-17A (0.39 pg/mL), TNF-α (0.61 pg/mL), and G-CSF (0.49 pg/mL). **b**, Representative flow cytometry plots and the number of Ly6C^+^ moDCs and neutrophils in the lung of naïve C57BL/6J mice (D0, n = 7); spike protein treated i.n. mice (D2^S^, n = 8); spike adjuvanted with 0.4 pg (D2^S+LPS 0.4 pg^, n = 10) or 1000 pg (D2^S+LPS 1000 pg^, n = 8) LPS treated i.n. mice at day 2 post intranasal administration analyzed by flow cytometry. **c**, Schematic of the experimental setup showing mRNA-LNP i.m. primed C57BL/6J mice were i.n. boosted with an unadjuvanted SARS-CoV-2 spike protein or with an endotoxin-removed SARS-CoV-2 spike protein at day 14 post intramuscular priming, followed by lung, BALF, and serum collection at day 7 post intranasal boosting. **d**, Number of extravascular IV^–^CD44^+^Tet^+^CD4^+^ T cells, IV^–^CD44^+^Tet^+^CD8^+^ T cells, IV^–^RBD^+^CD38^-^GL7^+^ GC-like B cells, IV^–^RBD^+^CD38^+^GL7^-^IgM^-^IgD^-^ CS B cells, IV^–^IgA^+^ ASCs, and IV^–^RBD^+^IgA^+^ ASCs in the lung of naïve mice (D0, n = 4); i.m. primed+i.n. boosted mice (D21^P+S^, n = 5); i.m. primed+i.n. endotoxin removed spike boosted mice (D21^P+S(removal)^, n = 5) at day 21 post-priming analyzed by flow cytometry as in **c**. **e**, ELISA measurements of SARS-CoV-2 S1-specific IgA and IgG in the BALF and serum from mice as in **d**. AUC, Area under the curve. Statistical significance was calculated by unpaired t-test (**a**) and one-way ANOVA (**b,d,e**). Tukey’s multiple comparisons were performed; *P < 0.05, **P < 0.01, ***P < 0.001, ****P < 0.0001. Data were pooled from two (**a,b**) independent experiments or representative (**d,e**) of an experiment. Values shown as zero indicate the absence of detectable cells (**b,d**). Source data

Lipopolysaccharide (LPS) triggers innate immune activation through the activation of the toll-like receptor-4, resulting in the accumulation of proinflammatory innate cells and cytokines in the lungs^27,28^. To rule out the possibility that LPS contamination during the preparation of the SARS-CoV-2 spike protein served as an adjuvant when using the spike protein as an intranasal booster, we measured the expression of several proinflammatory cytokines in the BALF of mice i.n. administered with the SARS-CoV-2 spike protein, and found that the expression of BALF interleukin-1α (IL-1α), IL-1β, IL-17A, tumor necrosis factor (TNF) and granulocyte–colony-stimulating factor (G-CSF) proteins was comparable at day 2 after administration between intranasal spike protein-treated and naive mice (Extended Data Fig. 6a). To assess whether LPS would activate the lung-resident innate immune cells when i.n. administered, C57BL/6J mice were i.n. treated with SARS-CoV-2 spike protein along with 0.4 pg and 1,000 pg of LPS. Intranasal administration of spike protein with even a trace amount of LPS (0.4 pg) was sufficient to notably increase the number of Ly6C^+^ moDCs and CD11b^+^Ly6G^+^ neutrophils in the lung mucosa at day 2 after treatment, whereas spike protein alone did not induce any notable changes compared with naive mice (Extended Data Fig. 6b), suggesting that the spike protein that we used in the intranasal booster did not contain innate immune stimulants. Next, mice primed i.m. with mRNA–LNPs were boosted i.n. with spike protein either before or after LPS removal using an endotoxin removal kit (Extended Data Fig. 6c). We observed no substantial differences in the number of spike-specific T cells and RBD-specific B cells in the lung mucosa (Extended Data Fig. 6d), as well as S1 IgA and IgG in the BALF and serum in mice i.n. boosted with either type of spike protein (Extended Data Fig. 6e). Collectively, these data indicated that robust cellular and humoral immune responses in the lung mucosa after an unadjuvanted intranasal booster administration were not driven by LPS contamination and that the spike protein itself could be used as a safe intranasal vaccine booster.

### Pre-existing CD4^+^ T cells serve as natural adjuvants

**Fig. 5:**
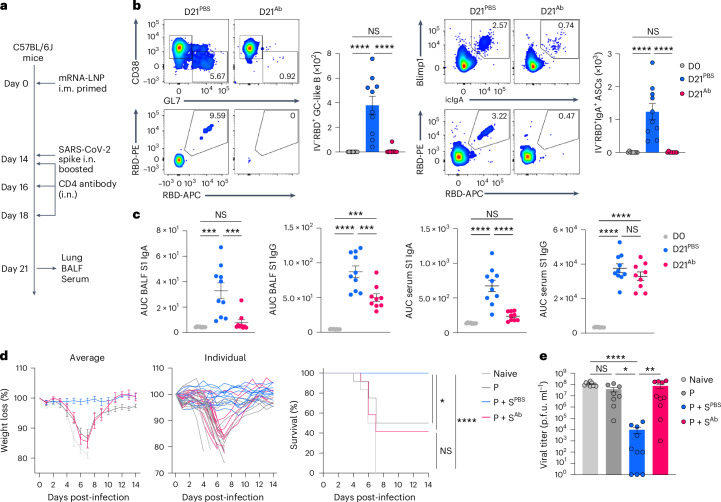
CD4^+^ T cells are indispensable for generating IgA recall response and providing protective mucosal immunity. **a**, Schematic of the experimental setup showing C57BL/6J mice primed i.m. with mRNA–LNPs at day 0, i.n. boosted with an unadjuvanted recombinant SARS-CoV-2 spike protein at day 14, followed by intranasal treatment with PBS or a CD4-depleting antibody at days 14, 16 and 18 after intramuscular priming and lung, BALF and serum collection at day 21 post-priming i.m. **b**, Representative flow cytometry plots and number of extravascular IV^−^RBD^+^CD38^−^GL7^+^ GC-like B cells and IV^−^RBD^+^IgA^+^ ASCs in the lungs of naive C57BL/6J mice (D0, *n* = 8); P + S mice treated with PBS (D21^PBS^, *n* = 10) or a CD4-depleting antibody (D21^Ab^, *n* = 9), analyzed by flow cytometry at day 21 as in **a**. **c**, ELISA measurements of SARS-CoV-2 S1-specific IgA and IgG in the BALF and serum from mice as in **b**. **d**, Weight loss and survival at days 0–14 post-infection with SARS-CoV-2 (WA1/2020) in naive K18-hACE2 mice (*n* = 6), prime-only mice (P, *n* = 9) and P + S mice treated with PBS (P + S^PBS^, *n* = 14) or CD4-depleting antibody (P + S^Ab^, *n* = 12) either i.n. or i.p. at days 14, 16, 18, 20, 23 and 26 post-priming i.m. and infected with SARS-CoV-2 (WA1/2020) at day 42 post-priming i.m. **e**, Lung viral titer in naive mice (*n* = 10); intramuscular prime-only mice (P, *n* = 8) and P + S mice treated with PBS (P + S^PBS^, *n* = 10) or a CD4-depleting antibody (P + S^Ab^, *n* = 10) as in **d** at day 2 post-infection with SARS-CoV-2 (WA1/2020) measured by plaque assay. Data are the mean ± s.e.m. The statistical significance was calculated using one-way ANOVA (**b**, **c**), log(rank) Mantel–Cox test (**d**) or nonparametric Kruskal–Wallis test (**e**). Tukey’s multiple comparisons (**b**, **c**), Bonferroni’s multiple comparisons (**d**) and Dunn’s multiple comparisons (**e**) were performed: *P < 0.05, **P < 0.01, ***P < 0.001, ****P < 0.0001. Data were pooled from two (**b**–**e**) independent experiments. Values indicated as zero represent the absence of detectable cells (**b**) or plaques (**e**). Source data

**Extended Data Fig. 7:**
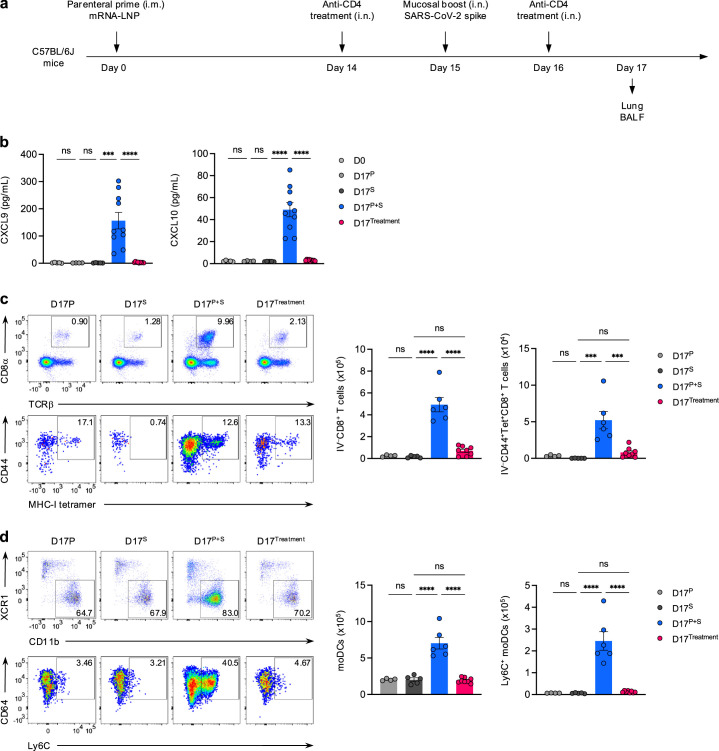
CD4^+^ T cells are essential for chemokine production and accumulation of CD8^+^ T cells and moDCs at the mucosal site. **a**, Schematic of the experimental setup showing C57BL/6J mice were i.m. immunized with mRNA-LNPs at day 0, i.n. boosted with an unadjuvanted SARS-CoV-2 spike protein at day 15, i.n. treated with PBS or a CD4 depleting antibody at days 14 and 16, followed by lung and BALF collection at day 2 post intranasal boosting. **b**, ELISA measurements of CXCL9 and CXCL10 concentrations in BALF of naïve C57BL/6J mice (n = 5); intramuscularprime-only mice (D17^P^, n = 4) and intranasalbooster-only mice (D17^S^, n = 5) at day 17 post-priming; P + S PBS-treated mice (D17^PBS^, n = 10); P + S CD4 depleting antibody-treated mice (D17^Ab^, n = 9) at day 17 post-priming as in **a**. Detection limits, CXCL9 (0.23 pg/mL) and CXCL10 (0.63 pg/mL). **c**, Representative flow cytometry plots and quantification of the number of extravascular IV^–^CD8^+^ T cells and IV^–^CD44^+^Tet^+^CD8^+^ T cells in the lung of intramuscular prime-only mice (D17^P^, n = 4) and intranasal booster-only mice (D17^S^, n = 5) at day 17 post-priming; P + S mice treated with PBS (D17^PBS^, n = 6) or CD4-depleting antibody (D17^Ab^, n = 8) at day 17 post priming as in **a. d**, Representative flow cytometry plots and quantification of the number of total and Ly6C^+^ moDCs in the lung from mice as in **c**. Mean ± SEM. Statistical significance was calculated by one-way ANOVA (**b-d**). Tukey’s multiple comparisons were performed; *P < 0.05, **P < 0.01, ***P < 0.001, ****P < 0.0001. Data were pooled from two (**b-d**) independent experiments. Values indicated as zero show the absence of detectable cells (**c,d**). Source data

CD4^+^ T cells provide local help for CD8^+^ T cell and B cell response against virus infection^29,30^, but their role in recall immunity after mucosal boosting remains unclear. To examine the role of pre-existing CD4^+^ T cells in lung immune cell accumulation within 2 d after boosting i.n., mRNA–LNP i.m. primed C57BL/6J mice were i.n. boosted with an unadjuvanted SARS-CoV-2 spike protein at day 15, followed by intranasal treatment of PBS or a CD4-depleting antibody at days 14 and 16 post-primary immunization (Extended Data Fig. 7a). At day 2 after boosting i.n. with spike protein, mice treated with an intranasal CD4 antibody had an almost complete loss of BALF CXCL9 and CXCL10 as well as lung CD44^+^Tet^+^CD8^+^ T cells and Ly6C^+^ moDCs compared with mice treated i.n. with PBS (Extended Data Fig. 7b–d), indicating an essential role of pre-existing CD4^+^ T cells in recruiting and activating the CD8^+^ T and innate immune cells into the lung. To test whether CD4^+^ T cells were also central for mucosal IgA recall response, C57BL/6J mice i.m. primed with mRNA–LNPs were i.n. boosted with an unadjuvanted SARS-CoV-2 spike protein at day 14 after priming i.m., followed by intranasal treatment with a CD4-depleting antibody at days 0, 2 and 4 after boosting i.n. (Fig. 5a). CD4 depletion led to a marked reduction in the number of RBD-specific, CD38^−^GL7^+^ GC-like B cells and IgA^+^ ASCs in the lung mucosa (Fig. 5b), as well as S1-specific IgA and IgG in the BALF, whereas systemic S1-specific IgG levels were unchanged compared with PBS treatment (Fig. 5c). Thus, CD4^+^ T_M_ cells were indispensable for anamnestic IgA responses in the lung mucosa.

To test whether pre-existing CD44^+^Tet^+^CD4^+^ T_M_ cells conferred protection, P + S K18-hACE2 mice, which express human angiotensin-converting enzyme 2 (ACE2) receptors mainly on epithelial cells, were injected i.n. and i.p. with PBS or a CD4-depleting antibody at days 0, 2, 4, 6, 9 and 12 after boosting i.n. with an unadjuvanted spike protein, followed by SARS-CoV-2 WA1/2020 virus infection at day 42 post-boosting i.n. Although i.n. boosted mice that were treated with PBS were protected against infection, as indicated by weight loss and survival, >50% of the mice treated with the CD4-depleting antibody succumbed at day 7 after viral infection (Fig. 5d) and had an increased infectious viral load in the lungs (Fig. 5e), indicating that loss of pre-existing CD4^+^ T cells eliminated the benefits of the intranasal booster. Thus, CD4^+^ T cells were required for the protective immunity conferred by the intranasal booster, at least in part by inducing respiratory IgA, IgG and tissue-resident CD8^+^ T_M_ cell responses.

### CD40 and TGFβ signaling drives lung IgA^+^ ASC differentiation

**Fig. 6:**
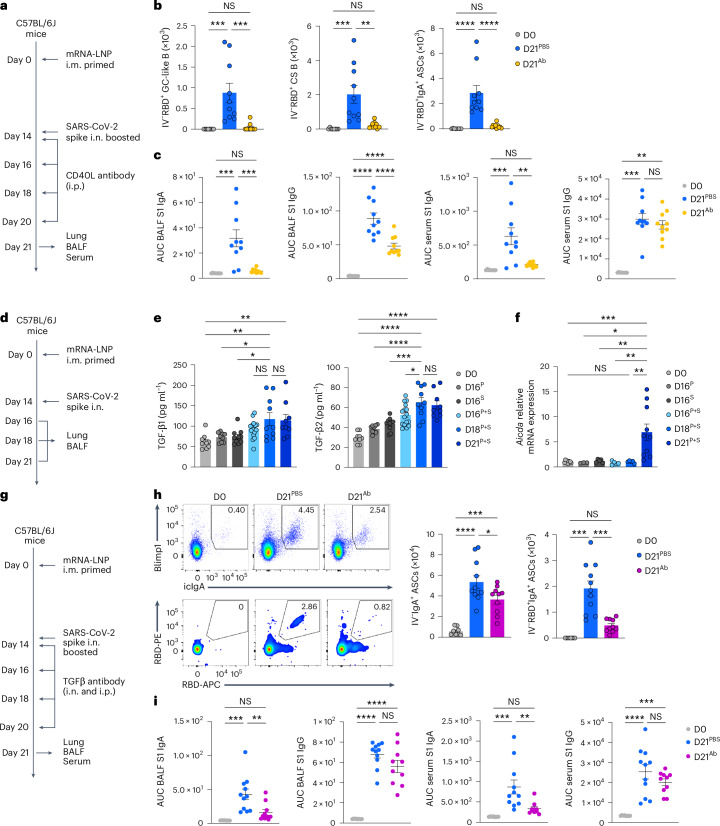
CD40 and TGFβ signaling induce the development of mucosal IgA recall responses. **a**, Schematic of the experimental setup showing C57BL/6J mice i.m. immunized with mRNA–LNPs at day 0, i.n. boosted with an unadjuvanted recombinant SARS-CoV-2 spike protein at day 14, followed by intraperitoneal treatment with PBS or a CD40L-blocking antibody at days 14, 16, 18 and 20 after intramuscular priming and lung, BALF and serum were analyzed at day 21 post-priming i.m. **b**, Number of extravascular IV^−^RBD^+^CD38^−^GL7^+^ GC-like B cells, IV^−^RBD^+^CD38^+^GL7^−^IgM^−^IgD^−^ class-switched B cells and IV^−^RBD^+^IgA^+^ ASCs in the lungs of naive C57BL/6J mice (D0, *n* = 8), P + S mice treated with PBS (D21^PBS^, *n* = 10) or CD40L-blocking antibody (D21^Ab^, *n* = 10), analyzed by flow cytometry at day 21 post-priming i.m. as in **a**. **c**, ELISA measurements of SARS-CoV-2 S1-specific IgA and IgG in the BALF and serum from mice as in **b**. **d**, Schematic of the experimental setup showing C57BL/6 mice primed i.m. with mRNA–LNPs at day 0 that were boosted i.n. with spike protein at day 14, followed by lung and BALF analysis at days 16, 18 and 21 after priming i.m. **e**, ELISA measurement of TGFβ1 and TGFβ2 in BALF from naive mice (D0, *n* = 10); intramuscular prime-only (D16^P^, n = 9) and intranasal booster-only (D16^S^, *n* = 10) at day 16, and P + S mice at day 16 (D16^P+S^, *n* = 16), day 18 (D18^P+S^, *n* = 10) and day 21 (D21^P+S^, *n* = 9) as in **d**. Detection limits for TGFβ1 and TGFβ2 were 2.44 pg ml^−1^. **f**, *Aicda* mRNA expression in purified CD19^+^ cells in the lungs of naive mice (D0, *n* = 9); intramuscular prime-only (D16^P^, *n* = 3) and intranasal booster-only mice (D16^S^, *n* = 5) at day 16, and P + S mice at day 16 (D16^P+S^, *n* = 5), day 18 (D18^P+S^, *n* = 5) and day 21 (D21^P+S^, *n* = 10) as in **d**, analyzed by quantitative PCR. Normalization of relative expression was to an average value obtained from the lungs of naive mice. **g**, Schematic of the experimental design showing C57BL/6 mice primed i.m. with mRNA–LNPs at day 0 and boosted i.n. with spike protein at day 14 that were i.p. and i.n. treated with PBS or a TGFβ-neutralizing antibody at days 14, 16, 18 and 20 post-boosting i.n., followed by analysis of lung, BALF and serum at day 21 post-priming i.m. **h**, Representative flow cytometry plots and quantification of extravascular total and RBD-specific IV^−^IgA^+^ ASC numbers in the lungs of D0 mice (*n* = 8) and D21^PBS^ mice (*n* = 11) or D21^Ab^ mice (*n* = 10) at day 21 post-priming as in **g**, analyzed by flow cytometry. **i**, ELISA measurements of SARS-CoV-2 S1-specific IgA and IgG in the BALF and serum from mice as in **h**. Data are the mean ± s.e.m. The statistical significance was calculated using one-way ANOVA (**b**,**c**,**e**,**f**,**h**,**i**). Tukey’s multiple comparisons (**b**,**c**,**e**,**f**,**h**,**i**) were performed: *P < 0.05, **P < 0.01, ***P < 0.001, ****P < 0.0001. Data were pooled from two (**b**,**c**,**f**,**h**,**i**) and three (**e**) independent experiments. Values shown as z**e**ro represent the absence of detectable cells (**b**,**h**). Source data

**Extended Data Fig. 8:**
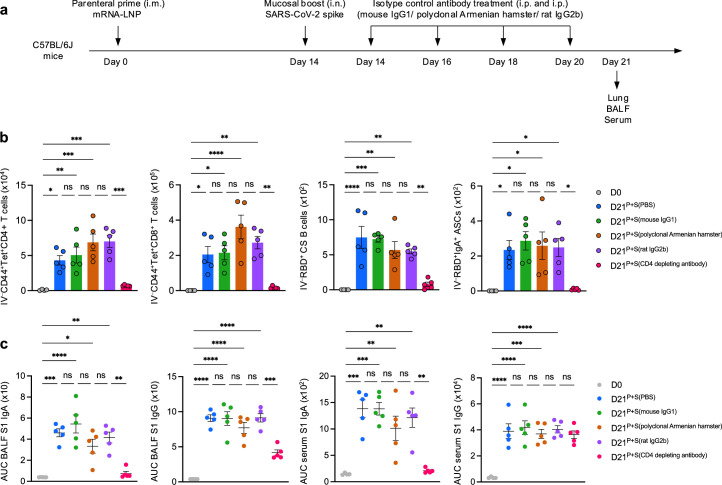
Mucosal recall responses were comparable between PBS-treated and isotype control antibody-treated P+S mice. **a**, Schematic of the experimental setup showing C57BL/6J mice were primed i.m. with mRNA-LNPs at day 0, i.n. boosted with an unadjuvanted SARS-CoV-2 spike protein at day 14, followed by intranasal and intraperitoneal treatment with PBS, mouse IgG1, polyclonal American hamster, rat IgG2b isotype antibodies, and a CD4 depleting antibody at days 0, 2, 4, and 6 post intranasal boosting, followed by lung, BALF, serum collection at day 7 post intransal booster. **b**, Number of extravascular CD44^+^Tet^+^CD4^+^ T cells, CD44^+^Tet^+^CD8^+^ T cells, RBD^+^CD38^+^GL7^-^IgM^-^IgD^-^ CS B cells, and RBD^+^IgA^+^ ASCs in the lung of naïve C57BL/6J mice (D0, n = 4); P + S mice treated with PBS (D21^PBS^, n = 5), mouse IgG1 antibody (D21^mouse IgG1^, n = 5), polyclonal Armenian hamster IgG antibody (D21^polyclonal Armenian hamster^, n = 5), rat IgG2b antibody (D21^rat IgG2b^, n = 5); and CD4 depleting antibody (D21^CD4^, n = 5) at day 21 post-priming analyzed by flow cytometry as in **a**. **c**, ELISA measurements of SARS-CoV-2 S1-specific IgA and IgG in the BALF and serum from mice as in **b**. AUC, Area under the curve. Mean ± SEM. Statistical significance was calculated by one-way ANOVA, and Tukey’s multiple comparisons (**b,c**) were performed; *P < 0.05, **P < 0.01, ***P < 0.001, ****P < 0.0001. Data were representative of an experiment (**b,c**). Values shown as zero indicate the absence of detectable cells (**b**). Source data

We next investigated the factors required for the antigen-specific IgA^+^ plasma cell development in the lower respiratory mucosa. To test whether CD40-mediated cognate interactions between CD4^+^ T cells and B cells in a TGFβ-rich niche were required for antigen-specific IgA^+^ ASC development, we injected P + S C57BL/6J mice with a CD40L-blocking antibody at days 0, 2, 4 and 6 post-boosting i.n. (Fig. 6a). Lung RBD-specific, CD38^−^GL7^+^ GC-like B cells, CD38^+^GL7^−^IgM^−^IgD^−^ class-switched B cells and IgA^+^ ASCs were nearly absent (Fig. 6b) and BALF S1-specific IgA and IgG were substantially reduced (Fig. 6c), whereas serum S1-specific IgG remained unchanged (Fig. 6c) in CD40L antibody-treated mice compared with PBS-treated mice, indicating that CD40 signaling was required for the development of antigen-specific IgA^+^ plasma cells after an intranasal booster. TGFβ1 and TGFβ2 were increased, and lung CD19^+^ B cells expressed *Aicda*, which is required for CSR^21–25^, in P + S mice compared with intramuscular prime-only and i.n. boosted-only mice at days 18 and 21 post-priming i.m. (Fig. 6d–f). Intranasal and intraperitoneal treatment of P + S C57BL/6J mice with a TGFβ-neutralizing antibody at 14, 16, 18 and 20 d post-boosting i.n. notably reduced the RBD-specific IgA^+^ ASC numbers in the lungs (Fig. 6g,h), and specifically decreased the S1-specific IgA, but not S1-specific the IgG, levels in the BALF and serum compared with PBS-treated P + S mice (Fig. 6i). All isotype control antibodies corresponding to CXCR3, CXCL9, CXCL10, CD4, CD40L and TGFβ antibody depletion experiments did not change the number of CD44^+^Tet^+^CD4^+^ T cells, CD44^+^Tet^+^CD8^+^ T cells, RBD^+^CD38^+^GL7^−^IgM^−^IgD^−^ class-switched B cells and RBD^+^IgA^+^ ASCs in the lung, and S1-specific IgA and IgG levels in the BALF and serum, compared with PBS treatment (Extended Data Fig. 8a–c), indicating that P + S mice treated with isotype control antibodies had comparable mucosal recall response to P + S mice treated with PBS. Thus, primed B cells developed into antigen-specific IgA^+^ ASCs with the help of CD40 and TGFβ stimulation on intranasal boosting with an unadjuvanted spike protein.

### Repeated nasal boosters enhance mucosal IgA responses

**Fig. 7:**
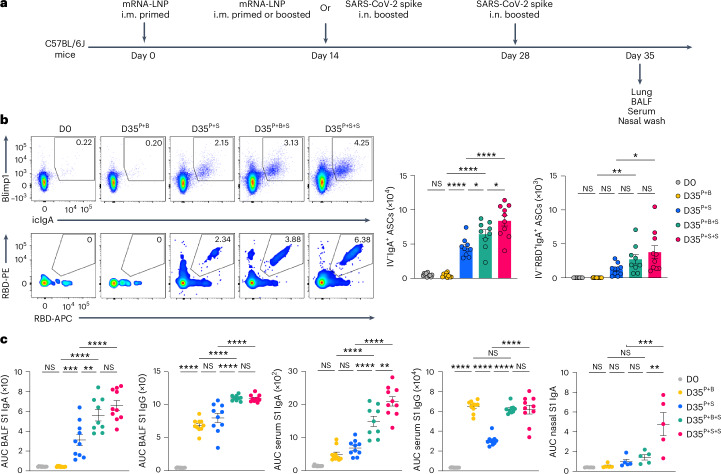
Repeated unadjuvanted nasal boosters enhance IgA recall responses in both upper and lower respiratory mucosa. **a**, Schematic of the experimental strategy showing C57BL/6J mice i.m. primed with mRNA–LNPs at day 0, i.m. boosted with mRNA–LNPs or i.n. boosted with an unadjuvanted recombinant SARS-CoV-2 spike protein at day 14, followed by boosting i.n. with homologous spike protein at day 28 post-prime, followed by lung, BALF, serum and nasal wash analysis at day 35 post-priming i.m. **b**, Representative flow cytometry plots and number of extravascular total and RBD-specific IV^−^IgA^+^ ASCs in the lungs of naive C57BL/6 mice (D0, *n* = 11), i.m. primed + i.m. boosted mice (D35^P+B^, *n* = 10), i.m. primed + i.n. spike boosted mice (D35^P+S^, *n* = 10), i.m. primed + i.m. boosted + i.n. boosted mice (D35^P+B+S^, *n* = 9) and i.m. primed + i.n. boosted + i.n. boosted mice (D35^P+S+S^, *n* = 10) at day 35 post-priming, analyzed by flow cytometry as in **a**. **c**, ELISA measurements of SARS-CoV-2 S1-specific IgA and IgG in the BALF, serum and nasal wash from mice as in **b**. Data are the mean ± s.e.m. The statistical significance was calculated using one-way ANOVA (**b**,**c**). Tukey’s multiple comparisons were performed: *P < 0.05, **P < 0.01, ***P < 0.001, ****P < 0.0001. Data were pooled from two (**b**,**c**) independent experiments. Values shown as zero indicate the absence of detectable cells (**b**). Source data

To investigate whether repeated exposure i.n. to antigen enhanced the lung mucosa IgA recall response, C57BL/6J mice i.m. primed with mRNA–LNPs were i.m. boosted with mRNA–LNPs or i.n. boosted with an unadjuvanted SARS-CoV-2 spike protein at day 14 post-priming, followed by intranasal boosting with an unadjuvanted, homologous, SARS-CoV-2 spike protein at day 14 post-boosting i.m. or i.n. We compared mucosal anamnestic IgA responses among P + S mice, i.m. primed + i.m. boosted (hereafter P + B) mice, i.m. primed + i.m. boosted + i.n. boosted (hereafter P + B + S) mice and i.m. primed + i.n. boosted + i.n. boosted (hereafter P + S + S) mice (Fig. 7a). At day 35 after intramuscular primary immunization, the number of total and RBD-specific IgA^+^ ASCs in the lung (Fig. 7b) and the amount of BALF and serum S1 IgA and IgG (Fig. 7c) were substantially higher in P + S + S mice than in P + S mice. Notably, the titer of nasal mucosa S1-specific IgA, which is key to preventing transmission^2,3,8^, was markedly higher in P + S + S mice compared with P + S and P + B + S mice (Fig. 7c). The number of lung IgA^+^ ASCs and the levels of BALF S1-specific IgA and IgG were also higher in P + B + S mice compared with P + B and P + S mice, although the amount of S1-specific IgA in lung mucosa was somewhat lower than that seen in P + S + S mice (Fig. 7b,c). These observations indicated that a second intranasal booster with spike protein further enhanced antigen-specific IgA responses in both the upper and the lower respiratory mucosa compared with a single intranasal booster.

## Discussion

In this study, we showed that boosting i.n. with an unadjuvanted protein generated microenvironmental niches that promoted the recruitment of primed lymphocytes from peripheral lymph nodes into the lung. In response to boosting i.n., these migrant memory B cells then differentiated into IgA^+^ plasma cells with help from memory CD4^+^ T cells. An unadjuvanted antigen delivered i.n. could redirect existing lymph node-resident memory B cells into the respiratory mucosa, whereby CD4^+^ T cells induced CXCL9 and CXCL10 recruited CD8^+^ T memory cells to establish a local tissue-resident CD8^+^ T_M_ cell pool and mediated B_M_ cell class switching and differentiation into local IgA-secreting plasma cells.

Our findings indicated that an unadjuvanted protein intranasal booster converted systemic immunity into robust recall responses at mucosal sites by harnessing lymph node-resident memory cells and utilizing CD4^+^ T cells as a natural adjuvant. This approach differs from chronic respiratory virus infection models^31,32^ or adjuvanted nasal vaccinations, which may bypass pre-existing adaptive immunity and carry potential risks of adverse events^13,33^. Although we utilized P + S, the underlying principle is not limited to this particular protocol but would also apply to vaccinated hosts who would inhale low, noninfectious levels of viruses in the environment. Periodic exposure to viral antigens in the air may be important in maintaining tissue-resident memory and boosting local IgA levels^34–36^. However, the exact amount of virus titers required to elicit robust recall responses in the respiratory mucosa needs to be further investigated.

We described antigen-specific recall IgA responses developing in the respiratory mucosa on boosting i.n. in mice using a fixed boosting scheme. Syrian hamsters vaccinated with P + S also mounted a robust mucosal immune response, which resulted in reduced SARS-CoV-2 transmission from infected co-housed animals^7^. Unadjuvanted spike protein boosting i.n. at day 84 post-priming i.m. induced potent cellular and humoral immune responses in the lung^7^. As antigen-specific B cells are detected in the spleen at day 60 post-mRNA–LNP vaccination i.m.^17,18,37^, the spleen could also serve as a potential source of B_M_ cells that could migrate into the lung when the interval between priming and intranasal boosting is prolonged. Furthermore, the unadjuvanted intranasal spike protein booster elicited respiratory mucosal and systemic IgA and IgG responses up to day 56 after boosting i.n., highlighting the durability of this approach^7^. These observations would suggest that an intranasal booster with an unadjuvanted protein after an intramuscular vaccine strategy can induce a long-lived immune response and is not limited to the mouse model or a fixed boosting scheme. However, further optimization studies in nonhuman primates with varying boosting timepoints will be necessary to generalize these conclusions. Finally, the general mechanistic understanding presented in the present study could be leveraged to develop promising and safe mucosal vaccine strategies for previously vaccinated or infected individuals, while also providing a better definition of the correlates of protection against respiratory viral infections.

## Methods

### Mice

Female 6- to 8-week-old C57BL/6 (CD45.2^+^), congenic C57BL/6 (*B6.SJL-PtprcaPep3b/BoyJ*) (*B6.Ly5.1*) (*CD45.1*^+^)*, Aicda-*ERT2-cre (B6.129P2-Aicda^*tm1.1(cre/ERT2)Crey*^/J), Rosa26-stop-tdTomato (*B6.Cg-Gt(ROSA)26Sor*^*tm14(CAG-tdTomato)Hze*^*/*J)*, Prdm1*-EYFP (*B6.Cg-Tg(Prdm1-EYFP)1Mnz/*J) and K18-hACE2 (*B6.Cg-Tg(K18-ACE2)2Prlmn/*J) mice were purchased from the Jackson Laboratory and *S1pr2*-ERT2-cre mice generated by T. Kurosaki and T. Okada were kindly provided by V. Gabriel at Rockefeller University. All mice were subsequently bred and housed at Yale University. All procedures used in the present study complied with federal guidelines and the institutional policies of the Yale School of Medicine Animal Care and Use Committee. No statistical methods were used to predetermine the sample size, but our sample sizes were similar to those of a previous publication^7^. Investigators were not blinded to allocation and age-matched animals were randomly assigned to experimental groups at the start of the experiments.

### SARS-CoV-2 infection and virus titer

As previously described^7^, Vero E6 cells overexpressing ACE2 and TMPRSS2 were cultured in Dulbecco’s modified Eagle medium (DMEM) supplemented with 1% sodium pyruvate and 5% fetal bovine serum (FBS) at 37 °C and 5% CO_2_. The SARS-CoV-2 isolate hCOV-19/USAWA1/2020 (NR-52281) was obtained from BEI Resources and amplified in Vero E6 cells overexpressing ACE2 and TMPRSS2. Cells were infected at a multiplicity of infection of 0.01 for 40–42 h. After incubation, the supernatant was collected and filtered through a 0.45-mm filter and stored at −80 °C. Viral titers were measured by plaque assay. Mice were anesthetized by 30% isoflurane (v:v) diluted in propylene glycol and i.n. infected with 6 × 10^4^ plaque-forming units (p.f.u.) of SARS-CoV-2. All procedures for propagation and infection of SARS-CoV-2 were performed in a BSL-3 facility with approval from the Yale Institutional Animal Care and Use Committee and Yale Environmental Health and Safety. For the quantification of SARS-CoV-2 titers, a plaque assay was conducted using Vero E6 cells overexpressing ACE2 and TMPRSS2. Plaques were visualized 48 h after infection through formalin fixation, Crystal Violet staining and water rinsing.

### Vaccination

As previously described^7^, the vials used for the Comirnaty vaccine were acquired from Yale Health pharmacy within 24 h of opening and stored at 4 °C. Vials contained residual vaccine (diluted to 100 μg ml^−1^ per manufacturer’s instructions), which was removed with a spinal syringe and pooled. The pooled residual vaccine was aliquoted and stored at −80 °C. SARS-CoV-2 spike protein (ACRO biosystems, cat. no. SPN-C52H9) was reconstituted in sterile endotoxin-free water according to the manufacturer’s protocol and then diluted in sterile PBS and stored at −80 °C. Endotoxin levels in spike protein were <0.05 EU per μg, as determined by the Limulus amebocyte lysate assay using the Endosafe Nexgen-PTS (Charles River Laboratories), indicating that this recombinant protein is of high purity. The effect of reconstituted spike protein used for the nasal booster on mucosal recall responses was further validated by removing endotoxin with the Proteus Endotoxin Removal Kit (BioRad) according to the manufacturer’s instructions. The mRNA–LNP vaccine encoding full-length SARS-CoV-2 spike protein (Pfizer/BioNTech BNT162b2) was diluted in sterile PBS and 50 μl was i.m. injected with a 31G syringe for a final dose of 0.05 μg or 1 μg. For P + S vaccination, 1 μg of mRNA–LNP vaccine was used. For the SARS-CoV-2 virus infection and plaque assay experiments, 0.05 μg of mRNA–LNP vaccine was used. For spike boosting, mice were anesthetized using a mixture of ketamine (100 mg per kg of body weight) and xylazine (10 mg per kg of body weight) and administered 1 μg of an unadjuvanted SARS-CoV-2 spike protein i.n. (in 20 μl of PBS), i.t. (in 20 μl of PBS) or i.p. (in 100 μl of PBS).

### Parabiosis

Parabiosis experiments were performed as previously described^16^. In brief, naive CD45.1 or primed CD45.2 C57BL/6 mice were anesthetized using a mixture of ketamine (100 mg per kg of body weight) and xylazine (10 mg per kg of body weight). After shaving the corresponding lateral aspects of each mouse, matching skin incisions were made from elbow joint to knee joint. Both olecranons and knee joints were connected and tied with absorbable 5/0 Vicryl sutures (Ethicon). After the attachment of the joints, the skin incision of each parabiont was clipped with 9-mm stainless steel wound clips (Fine Science Tools).

### In vivo treatment

For FTY720 treatment, mRNA–LNP i.m. primed mice were given 50 μg of FTY720 (Sigma-Aldrich) i.p. every other day, starting at days 0, 2 or 4 post-boosting i.n. To block the CXCR3–CXCL9 and CXCR3–CXCL10 signaling axis, mRNA–LNP i.m. primed mice were i.p. injected with 500 μg of CXCR3-, CXCL9- and CXCL10-blocking antibodies (BioXCell) at days 14, 16, 18 and 20. To deplete pulmonary CD4 T cells, i.m. primed mice were i.n. administered 50 μg of CD4-depleting antibody (BioXCell) at days 14, 16 and 18. For pulmonary CD8^+^ T cells and moDC analysis, mRNA–LNP i.m. immunized mice were i.n. treated with 50 μg of CD4-depleting antibody (BioXCell) at days 14 and 16. To deplete CD4 T cells before SARS-CoV-2 infection, i.m. primed K18-hACE2 mice were i.p. and i.n. injected with 300 μg and 100 μg of CD4-depleting antibody at days 14, 16, 18, 20, 23 and 26 post-priming i.m. To block the CD40L signaling, i.m. primed mice were i.p. treated with 400 μg of CD40L-blocking antibody (BioXCell) at days 14, 16, 18 and 20 post-priming. For TGFβ neutralization, i.m. primed mice were i.p. and i.n. treated with 600 μg and 200 μg of TGFβ-neutralizing antibody (BioXCell), respectively. Route and dose for mouse IgG1 (BioXCell), polyclonal Armenian hamster (BioXCell) and rat IgG2b (BioXCell) isotype control antibodies were used for the corresponding depletion assays. For fate mapping of GC B cells, primed *S1pr2*-tdTomato mice were i.p. injected with 2 mg of 4-hydroxytamoxifen (Sigma-Aldrich) at either days 6, 7, 8, 9 and 10 or days 34, 35, 36, 37, 38 and 39. For imaging analysis, i.m. primed *Aicda*-tdTomato-*Prdm1*-EYFP reporter mice were given, by oral gavage, 200 μl of tamoxifen (Sigma-Aldrich) dissolved in corn oil (Sigma-Aldrich) at 50 mg ml^−1^ at days 4, 8 and 12 post-priming i.m. as previously described^38^. For endotoxin treatment, LPS (Invivogen, cat. no. R595) was reconstituted with endotoxin-free PBS at 2.5 mg ml^−1^ and stored at −80 °C; 0.4 pg and 1,000 pg of LPS with 1 μg of recombinant spike protein per mouse were i.n. administered.

### Lung leukocyte isolation

Mice were i.v. injected with 2 μg of FITC-labeled, anti-CD45 antibody to discriminate extravascular leukocytes from circulating populations. After 3 min, mice were euthanized to harvest and analyze the tissues. Single-cell suspensions from the lungs were prepared as previously described^7,39^. In brief, lungs were minced with scissors and incubated in a digestion cocktail containing collagenase A (1 mg ml^−1^, Roche) and DNase I (30 μg ml^−1^, Sigma-Aldrich) in PBS at 37 °C for 45 min under gentle agitation, followed by filtering with a 70-μm mesh. After centrifugation at 600*g* for 3 min and 4 °C, pellets were treated with ammonium–chloride–potassium buffer to lyse red blood cells at room temperature for 2 min and washed with FACS buffer (PBS with 3% FBS). After centrifugation, pellets were resuspended with FACS buffer for downstream analysis.

### Flow cytometry

Single-cell suspensions were stained with multiparameter antibodies and analyzed using an LSR II flow cytometer (Becton Dickinson). For spike-specific T analysis, single-cell suspensions from the lung were stained with allophycocyanin (APC)-labeled SARS-CoV-2 62-76 MHC-II tetramer (I-A(b)) for 1 h at room temperature. After washing with FACS buffer, cells were stained with other surface makers including phycoerythrin (PE)-labeled SARS-CoV-2 539-546 MHC-I tetramer (H-2K(b)) for 20 min at 4 °C. For RBD-specific B cell analysis, RBD tetramers were produced by incubating recombinant SARS-CoV-2 spike RBD His Biotin Protein, CF (R&D, cat. no. BT10500-050) with either streptavidin–PE (Prozyme, cat. no. PJRS25) or streptavidin–APC (Prozyme, cat. no. PJ27S) at a 4:1 molar ratio for ON at 4 °C (ref. ^7^). Single-cell suspensions were stained with RBD tetramers and other surface markers for 20 min at 4 °C. To detect RBD-specific, IgA-secreting plasma cells, surface-stained cells were washed with FACS buffers, followed by fixation (Invitrogen) for 30 min at 4 °C. Fixed cells were intracellularly stained with BLIMP1 and IgA for 30 min at room temperature as previously described^16^. BD FACS Diva software (v.9.3.1) and FlowJo (v.10.9.0) were used for data collection and analysis.

### Antibodies

For surface staining, anti-CD45 (clone 30-F11, 1:200), anti-B220 (clone RA3-6B2, 1:1000), anti-CD19 (clone ID3, 1:200), anti-IgM (clone II/41, 1:200), anti-IgD (clone 11-26 c.2a, 1:1,000), anti-CD38 (clone 90, 1:200), anti-GL7 (clone GL7, 1:200), anti-CD138 (clone 281-2, 1:200), anti-TCRβ (clone H57-597, 1:200), anti-CD4 (clone GK1.5, 1:200), anti-CD8α (clone 53-6.7, 1:200), anti-CD69 (clone H1.2F3, 1:200), anti-CD103 (clone 2E7, 1:200), anti-PD-1 (clone 29F.1A12, 1:200), anti-CD44 (clone IM7, 1:100), anti-CD11b (clone M1/70, 1:1,000), anti-CD11c (clone N418, 1:1,000), anti-CD64 (clone X54-5/7.1, 1:1,000), anti-MHC-II (clone M5/114.15.2, 1:1,000), anti-Siglec-F (clone E50-2440, 1:1,000), anti-Ly6C (clone HK1.4, 1:200), anti-Ly6G (clone 1A8, 1:200), anti-NK1.1 (clone PK136, 1:200) and anti-XCR1 (clone ZET, 1:1000) antibodies were purchased from BioLegend, BD Biosciences and Invitrogen. For chemokine receptor staining, anti-CXCR3 (clone CXCR3-173, 1:100), anti-CXCR5 (clone 2G8, 1:50) and anti-CXCR6 (clone SA051D1, 1:100) antibodies were purchased from BioLegend and BD Biosciences. For intracellular staining, anti-BLIMP1 (clone 5E7, 1:100) and anti-IgA (clone C10-1, 1:200) antibodies were purchased from BD Biosciences. Alexa Fluor-488-conjugated donkey anti-mouse IgG (H + L) antigen-binding fragment (Fab; 1:1,000) was purchased from Jackson ImmunoResearch Laboratories. For immunofluorescence analysis, Alexa Fluor-647-conjugated, polyclonal anti-IgA antibody (1:400) was purchased from SouthernBiotech.

### ELISA

Ninety-six-well plates (Thermo Fisher Scientific, cat. no. 442404) were coated with recombinant SARS-CoV-2 S1 protein (ACRO Biosystems, cat. no. S1N-C52H3). After overnight incubation at 4 °C, the plates were washed with PBS-T (PBS with 0.05% Tween-20) and blocked with blocking solution (PBS with 0.1% Tween-20 and 3% milk powder) for 1–2 h at room temperature. BALF or serum samples diluted with dilution solution (PBS with 0.1% Tween-20 and 1% milk powder) were added to the wells and incubated for 2 h at room temperature. After washing more than 5× with PBS-T using an automatic plate washer, horseradish peroxidase (HRP)-conjugated anti-mouse IgG (Cell Signaling Technology, cat. no. 7076, 1:3,000) and HRP anti-mouse IgA (SouthernBiotech, cat. no. 1040-05, 1:1,000) were added to the plates for 2 h at room temperature, followed by washing and adding TMB Substrate Reagent Set (BD Biosciences, cat. no. 555214). The reaction was stopped with 2N sulfuric acid after 10 min of substrate development, then absorbance was measured at wavelengths of 450 nm. For cytokine and chemokine measurement, BALF was shipped to Eve Technologies on dry ice and analyzed using the following panels: Mouse Cytokine 32-plex Discovery Assay (MD32) and Multi-Species TGFβ 3-plex Discovery Assay (TGFβ1–TGFβ3).

### B cell isolation and qPCR

Preparation of single-cell suspensions from the lung has been described above. B cells were isolated from lung single-cell suspensions using EasySep Mouse B cell Isolation Kit (STEMCELL Technologies), following the manufacturer’s instructions. TRIzol (Invitrogen) and Quantitect reverse transcription kit (QIAGEN) were used for RNA isolation and complementary DNA synthesis, according to the manufacturer’s instructions. Quantitative PCR (qPCR) was carried out using SYBR Green-based quantification (BioRad). The messenger RNA expression of *Aicda* was measured by reverse transcription qPCR (RT–qPCR). Expression was normalized against the housekeeping gene *Hprt*. The following set of primers was used: *Aicda* (5′-GGCTGAGGTTAGGGTTCCATCTCAG-3′ and 5′-GAGGGAGTCAAGAAAGTCACGCTGGA-3′)^40^ and *Hprt* (5′-AGTGTTGGATACAGGCCAGAC-3′ and 5′-CGTGATTCAAATCCCTGAAGT-3′).

### Immunofluorescence

Mice were euthanized and perfused with PBS and 1% paraformaldehyde (PFA) diluted in PBS via intracardiac injection. Lung tissues were harvested and fixed with 1% PFA overnight at 4 °C. For cryoprotection, lung tissues were immersed in 30% sucrose overnight at 4 °C. Lung lobes were embedded in an optimal cutting temperature (OCT) compound (Sakura Finetek) and then frozen with acetone and dry ice. Then, 15-μm sections of lung tissue were cut on a cryostat. Sections were washed with PBS to remove OCT and blocked with a solution of 1% bovine serum albumin, glycine (22 mg ml^−1^) and 0.1% (v:v) Tween-20 for 1 h at room temperature. Samples were incubated with AF647-conjugated, anti-IgA antibody (SouthernBiotech, 1:400) in a humidified chamber overnight at 4 °C. After washing 3× with PBS, 0.3% Sudan Black B solution in 70% ethyl alcohol was added to samples for 10 min at room temperature. Slides were washed with PBS 3× and immersed in distilled water. Slides were mounted with a solution of DAPI Prolong Gold antifade reagent (Invitrogen) with 17.5 mg ml^−1^ of DAPI and covered with a coverslip. Images were acquired using the Stellaris Confocal microscope (Leica) and LAS X software (v.5.3.0). ImageJ was used for further image analysis.

### scRNA-seq and data analysis

C57BL/6J mice were i.m. immunized with 1 μg of mRNA–LNP vaccine, followed by boosting i.n. with either PBS or 5 μg of spike protein post-priming i.m. At day 2 after an intranasal booster, single cells from lung tissue were isolated and tissue-resident CD45^+^ immune cells were sorted. Single-cell suspensions of sorted cells were loaded on to the Chromium Controller (10x Genomics) for droplet formation. ScRNA-seq libraries were generated using the Chromium Single Cell 3′ Reagent Kit (10x Genomics) according to the manufacturer’s protocol. Sample libraries were sequenced on the Illumina Novaseq 6000. Sequencing results were converted to FASTQ sequences and mapped to the GRCm38 mouse reference genome using the cellranger (v.3.1.0) mkfastq function. A gene-by-cell unique molecular identifier (UMI) count matrix was generated with default settings. Cells containing more than a high percentage of mitochondrial genes (>10%) and low UMI counts (<1,000) were filtered out using the addPerCellQC function of scater R package (v.1.15.6). Doublet cells were also excluded using the scDblFinder function of the scDblFinder R package. Quality control-positive cells were clustered into 16 clusters using the FindClusters functions of the Seurat R package, with the first 15 principal components of highly variable genes and resolution = 0.5. Cell clusters were visualized in the two-dimensional Uniform Manifold Approximation and Projection (UMAP) plot using the RunUMAP function of the Seurat R package (v.3.1.5). Cell types were determined based on immune cell markers using previously published papers and databases such as immgen.org. Differentially expressed genes (DEGs) between prime-only and prime and spike vaccination conditions were defined using the FindMarkers function of Seurat R package. For GO term analysis, gene sets were downloaded genome-wide by annotation for mouse using the org.Mm.eg.db (v.3.15) R package. Upregulated DEGs in prime and spike compared with prime-only conditions were used to show the top 15 GO terms.

### Statistical analysis

Prism software (GraphPad, v.10) was used for visualization and statistical analysis. Survival curves were analyzed using the log(rank) Mantel–Cox test, followed by Bonferroni’s multiple comparisons. Others were analyzed using unpaired Student’s *t*-test and ordinary one-way analysis of variance (ANOVA) or the nonparametric Kruskal–Wallis test was used based on the Gaussian distribution test by generating a quantile–quantile plot. Tukey’s or Dunn’s multiple comparisons were consistently performed. No data were excluded from the analyses.

### Reporting summary

Further information on research design is available in the Nature Portfolio Reporting Summary linked to this article.

## Online content

Any methods, additional references, Nature Portfolio reporting summaries, source data, extended data, supplementary information, acknowledgements, peer review information; details of author contributions and competing interests; and statements of data and code availability are available at 10.1038/s41590-025-02156-0.

## Supplementary information

Reporting Summary

## Source data

Source Data Statistical source data for Figs. 1–7 and Extended Data Figs. 2–4 and 6–8.

## Data Availability

The scRNA-seq data in the present study have been deposited in the Sequence Read Archive (SRA) database of BioProject under accession no. PRJNA1240280. Source data are provided with this paper.
