# Supplementary material for: Mucosal unadjuvanted booster vaccines elicit local IgA responses by conversion of pre-existing immunity in mice
Source: Nat Immunol. 2025 May 13;26(6):908–19. doi: 10.1038/s41590-025-02156-0 (PMC12133566; doi:10.1038/s41590-025-02156-0)
Supplement: Supplementary file 1 — Reporting Summary [file 41590_2025_2156_MOESM1_ESM.pdf]

nature portfolio

Corresponding author(s): Akiko Iwasaki

Last updated by author(s): Apr 2, 2025

Reporting Summary

Nature Portfolio wishes to improve the reproducibility of the work that we publish. This form provides structure for consistency and transparency in reporting. For further information on Nature Portfolio policies, see our Editorial Policies and the Editorial Policy Checklist.

Statistics

For all statistical analyses, confirm that the following items are present in the figure legend, table legend, main text, or Methods section.

n/a	Confirmed	
☐	☒	The exact sample size (*n*) for each experimental group/condition, given as a discrete number and unit of measurement
☐	☒	A statement on whether measurements were taken from distinct samples or whether the same sample was measured repeatedly
☐	☒	The statistical test(s) used AND whether they are one- or two-sided<br>*Only common tests should be described solely by name; describe more complex techniques in the Methods section.*
☒	☐	A description of all covariates tested
☐	☒	A description of any assumptions or corrections, such as tests of normality and adjustment for multiple comparisons
☐	☒	A full description of the statistical parameters including central tendency (e.g. means) or other basic estimates (e.g. regression coefficient) AND variation (e.g. standard deviation) or associated estimates of uncertainty (e.g. confidence intervals)
☐	☒	For null hypothesis testing, the test statistic (e.g. *F*, *t*, *r*) with confidence intervals, effect sizes, degrees of freedom and *P* value noted<br>*Give P values as exact values whenever suitable.*
☒	☐	For Bayesian analysis, information on the choice of priors and Markov chain Monte Carlo settings
☒	☐	For hierarchical and complex designs, identification of the appropriate level for tests and full reporting of outcomes
☒	☐	Estimates of effect sizes (e.g. Cohen's *d*, Pearson's *r*), indicating how they were calculated

Our web collection on statistics for biologists contains articles on many of the points above.

Software and code

Policy information about availability of computer code

Data collection	BD FACS Diva Software v9.3.1<br>Las X softward v5.3.0<br>10X Chromium 3'-mRNA library kit<br>HISEQ4000 (illumina sequencing)
Data analysis	FlowJo v10.9.0. GaphPad Prism v10.<br>CellRanger v3.1.0, DropletUtils (v1.61) R package, Scater (v1.15.6) R package, Scran (v1.14.6) R package, Seurat (v3.1.5) R package.

For manuscripts utilizing custom algorithms or software that are central to the research but not yet described in published literature, software must be made available to editors and reviewers. We strongly encourage code deposition in a community repository (e.g. GitHub). See the Nature Portfolio guidelines for submitting code & software for further information.

Data

Policy information about availability of data

All manuscripts must include a data availability statement. This statement should provide the following information, where applicable:

- Accession codes, unique identifiers, or web links for publicly available datasets
- A description of any restrictions on data availability
- For clinical datasets or third party data, please ensure that the statement adheres to our policy

The single-cell RNA sequencing (scRNA-seq) raw data in this study have been deposited in the SRA database of BioProject under the accession code PRJNA1240280. The processed meta data of scRNA-seq are available upon reasonable requests to the corresponding author. The source data are provided with this paper.

Research involving human participants, their data, or biological material

Policy information about studies with human participants or human data. See also policy information about sex, gender (identity/presentation), and sexual orientation and race, ethnicity and racism.

Reporting on sex and gender	n/a
Reporting on race, ethnicity, or other socially relevant groupings	n/a
Population characteristics	n/a
Recruitment	n/a
Ethics oversight	n/a

Note that full information on the approval of the study protocol must also be provided in the manuscript.

Field-specific reporting

Please select the one below that is the best fit for your research. If you are not sure, read the appropriate sections before making your selection.

☒ Life sciences ☐ Behavioural & social sciences ☐ Ecological, evolutionary & environmental sciences

For a reference copy of the document with all sections, see nature.com/documents/nr-reporting-summary-flat.pdf

Life sciences study design

All studies must disclose on these points even when the disclosure is negative.

Sample size	No statistical methods were used to pre-determine sample sizes but our sample sizes are similar to those of the previous publication (ref 7). We aimed to have at least 3 sample sizes in each group in each independent experiment to have statistical power.
Data exclusions	No data were excluded.
Replication	Experiments were repeated with at least twice for all results presented in the manuscript. Details are shown in the figure legends.
Randomization	Age and sex-matched animals were randomly assigned to experimental groups at the beginning of the experiments. For SARS-CoV-2 virus propagation and plaque assay experiments, random allocation was not applicable as all samples were derived from the same cell line under identical culture conditions.
Blinding	Since vaccination, treatment and experimental analysis could not be separated, all the investigators were not blinded.

Reporting for specific materials, systems and methods

We require information from authors about some types of materials, experimental systems and methods used in many studies. Here, indicate whether each material, system or method listed is relevant to your study. If you are not sure if a list item applies to your research, read the appropriate section before selecting a response.

Materials & experimental systems

n/a	Involved in the study
☐	☒ Antibodies
☐	☒ Eukaryotic cell lines
☒	☐ Palaeontology and archaeology
☐	☒ Animals and other organisms
☒	☐ Clinical data
☒	☐ Dual use research of concern
☒	☐ Plants

Methods

n/a	Involved in the study
☒	☐ ChIP-seq
☐	☒ Flow cytometry
☒	☐ MRI-based neuroimaging

Antibodies

Antibodies used

Antibody name/ Supplier name/ Clone name/ Catalog number/ Dilution/ Validation (manufacturer's website)
CD4 BD BiosciencesGK1.5 563790 1:200 https://www.bdbiosciences.com/en-us/products/reagents/flow-cytometry-reagents/research-reagents/single-color-antibodies-ruo/buv395-rat-anti-mouse-cd4.563790?tab=product_details
CD8 BD Biosciences53-6.7 563152 1:200 https://www.bdbiosciences.com/en-us/products/reagents/flow-cytometry-reagents/research-reagents/single-color-antibodies-ruo/bv605-rat-anti-mouse-cd8a.563152?tab=product_details
CD11b Invitrogen M1/70 A15390 1:1000 https://www.thermofisher.com/antibody/product/CD11b-Antibody-clone-M1-70-Monoclonal/A15390
CD11c BioLegend N418 117324 1:1000 https://www.biolegend.com/nl-be/products/apc-cyanine7-anti-mouse-cd11c-antibody-3931
CD19 BD Biosciences 1D3 562958 1:200 https://www.bdbiosciences.com/en-no/products/reagents/flow-cytometry-reagents/research-reagents/single-color-antibodies-ruo/bv510-rat-anti-mouse-cd19.562956?tab=product_details
CD38 Invitrogen 90 56-0381-82 1:200 https://www.thermofisher.com/antibody/product/CD38-Antibody-clone-90-Monoclonal/56-0381-82
CD44 BioLegend IM7 103026 1:100 https://www.biolegend.com/nl-nl/products/alexa-fluor-700-anti-mouse-human-cd44-antibody-3406
CD45 BioLegend 30-F11 103108 1:200 (1.5 ug for IV injection) https://www.biolegend.com/en-gb/products/fitc-anti-mouse-cd45-antibody-99
CD45R/B220 BD RA3-6B2 552772 1:1000 https://www.bdbiosciences.com/en-fi/products/reagents/flow-cytometry-reagents/research-reagents/single-color-antibodies-ruo/pe-cy-7-rat-anti-mouse-cd45r-b220.552772?tab=product_details
CD64 BioLegend X54-5/7.1 139306 1:1000 https://www.biolegend.com/de-de/products/apc-anti-mouse-cd64-fcgammari-antibody-7874
CD69 BD Biosciences H1.2F3 562455 1:200 https://www.bdbiosciences.com/en-lu/products/reagents/flow-cytometry-reagents/research-reagents/single-color-antibodies-ruo/pe-cf594-hamster-anti-mouse-cd69.562455?tab=product_details
CD103 BioLegend 2E7 121426 1:200 https://www.biolegend.com/de-at/products/pe-cyanine7-anti-mouse-cd103-antibody-9899
CD138 BD Biosciences 281-2 563147 1:200 https://www.bdbiosciences.com/en-us/products/reagents/flow-cytometry-reagents/research-reagents/single-color-antibodies-ruo/bv605-rat-anti-mouse-cd138.563147?tab=product_details
CD183(CXCR3) BD Biosciences CXCR3-173, 740630 1:100 https://www.bdbiosciences.com/en-us/products/reagents/flow-cytometry-reagents/research-reagents/single-color-antibodies-ruo/bv650-hamster-anti-mouse-cd183.740630?tab=product_details
CD185(CXCR5) BD Biosciences 2G8 562889 1:50 https://www.bdbiosciences.com/en-us/products/reagents/flow-cytometry-reagents/research-reagents/single-color-antibodies-ruo/bv421-rat-anti-mouse-cd185-cxcr5.562889?tab=product_details
CD186(CXCR6) BioLegend SA051D1 151111 1:100 https://www.biolegend.com/ja-jp/products/brilliant-violet-711-anti-mouse-cd186-cxcr6-antibody-15094
CD279(PD-1) Biolegend 29F.1A12 135225 1:200 https://www.biolegend.com/ja-jp/products/brilliant-violet-785-anti-mouse-cd279-pd-1-antibody-9874
TCR-beta BD Biosciences H57-597 5606566 1:200 https://www.bdbiosciences.com/en-us/products/reagents/flow-cytometry-reagents/research-reagents/single-color-antibodies-ruo/apc-cy-7-hamster-anti-mouse-tcr-chain.560656?tab=product_details
GL7 Invitrogen GL7 48-5902-82 1:200 https://www.thermofisher.com/antibody/product/GL7-Antibody-clone-GL-7-GL7-Monoclonal/48-5902-82
IgM Invitrogen II/41 46-5790-82 1:200 https://www.thermofisher.com/antibody/product/IgM-Antibody-clone-II-41-Monoclonal/41-5790-82
IgD BD Biosciences 11-26c.2a 5636188 1:1000 https://www.bdbiosciences.com/en-us/products/reagents/flow-cytometry-reagents/research-reagents/single-color-antibodies-ruo/bv786-rat-anti-mouse-igd.563618?tab=product_details
IgA BD C10-1 743297 1:200 https://www.bdbiosciences.com/en-us/products/reagents/flow-cytometry-reagents/research-reagents/single-color-antibodies-ruo/bv711-rat-anti-mouse-iga.743297?tab=product_details
Blimp1 BD 5E7 564269 1:100 https://www.bdbiosciences.com/en-us/products/reagents/flow-cytometry-reagents/research-reagents/single-color-antibodies-ruo/pe-cf594-rat-anti-mouse-blimp-1.564269?tab=product_details
MHC II BioLegend M5/114.15.2 107641 1:1000 https://www.biolegend.com/nl-nl/products/brilliant-violet-650-anti-mouse-i-a-i-e-antibody-12085
XCR1 BioLegend SET 148216 1:1000 https://www.biolegend.com/en-ie/products/brilliant-violet-421-anti-mouse-rat-xcr1-antibody-10750
SiglecF BD E50-2440 552126 1:1000 https://www.bdbiosciences.com/en-us/products/reagents/flow-cytometry-reagents/research-reagents/single-color-antibodies-ruo/pe-rat-anti-mouse-siglec-f.552126?tab=product_details
NK1.1 BioLegend PK136 108726 1:200 http://biolegend.com/en-gb/products/percp-anti-mouse-nk-1-1-antibody-4288?GroupID=GROUP20
Ly6C BioLegend HK1.4 128024 1:200 https://www.biolegend.com/ja-jp/products/alexa-fluor-700-anti-mouse-ly-6c-antibody-6757
Ly6G BioLegend 1A8 127645 1:200 https://www.biolegend.com/en-ie/products/brilliant-violet-785-anti-mouse-ly-6g-antibody-12245
InvivoMab CD4 BioXcell GK1.5 BE003-1 https://bioxcell.com/invivomab-anti-mouse-cd4-be0003-1
InvivoMab CD154(CD40L) BioXcell MR-1 BE0017-1 https://bioxcell.com/invivomab-anti-mouse-cd40l-cd154-be0017-1
InvivoMab CD183(CXCR3) BioXcell CXCR3-173 BE0249 https://bioxcell.com/invivomab-anti-mouse-cxcr3-cd183-be0249

	InvivoMab CXCL9(MIG) BioXcell MIG-2F5.5 BE0309 https://bioxcell.com/invivomab-anti-mouse-cxcl9-mig-be0309<br>InvivoMab CXCL10(IP-10) BioXcell 1F11 BE0440 https://bioxcell.com/invivomab-anti-mouse-cxcl10-ip-10-be0440<br>InvivoMab TGF-beta BioXcell 1D11.16.8 BE0057 https://bioxcell.com/invivomab-anti-mouse-human-rat-monkey-hamster-canine-bovine-tgf-beta-be0057<br>InvivoMab mouse IgG1 isotype control BioXcell unknown specificity BE0083 https://bioxcell.com/invivomab-mouse-igg1-isotype-control-unknown-specificity-be0083<br>InvivoMab polyclonal Armenian hamster IgG BE0091 https://bioxcell.com/invivomab-polyclonal-armenian-hamster-igg-be0091<br>InvivoMab rat IgG2b isotype control, anti-keyhole limpet hemocyanin BE0090 https://bioxcell.com/invivomab-rat-igg2b-isotype-control-anti-keyhole-limpet-hemocyanin-be0090<br>AF488 anti-mouse IgG(H+L) Fab Jackson ImmunoResesarch polyclonal 715-547-033 1:2000 https://www.jacksonimmuno.com/catalog/products/715-547-003<br>AF647 anti-mouse IgA SouthernBiotech polyclonal 1040-31 1:400 https://www.fishersci.com/shop/products/iga-goat-anti-mouse-alexa-fluor-647-polyclonal-southerbiotech/OB104031<br>HRP-anti-IgA SouthernBiotech polyclonal 1040-05 1:1000 https://www.southernbiotech.com/goat-anti-mouse-iga-hrp-1040-05<br>HRP-anti-IgG Cell Signaling Technology polyclonal 7076 1:3000 https://www.cellsignal.com/products/secondary-antibodies/anti-mouse-igg-hrp-linked-antibody/7076?srsltid=AfmBOoolrYDhocXhWyYrsrUz6qtuTuX1KLE66bPEBTLFTV__5csEC8qw
Validation	All antibodies are commercially available and have been validated by manufacturers and relevant references were cited on the manufacturer's website. Vendor websites for antibodies were listed above and the validations can be found there. All antibodies were tested in the laboratory using positive and negative controls and titrated before all experiments.

Eukaryotic cell lines

Policy information about cell lines and Sex and Gender in Research

Cell line source(s)	Vero E6 cells (ATCC CCL-81). Vero E6 cells overexpressing angiotensin-converting enzyme 2 (ACE2) and TMPRSS2 was kindly provided by B. Graham at the National Institutes of Health Vaccine Research Center (NIH-VRC).
Authentication	Cell lines were authenticated by morphology.
Mycoplasma contamination	Cell lines were negative for mycoplasma contamination.
Commonly misidentified lines<br>(See ICLAC register)	No misidentified cell lines in this study.

Animals and other research organisms

Policy information about studies involving animals; ARRIVE guidelines recommended for reporting animal research, and Sex and Gender in Research

Laboratory animals	Six to eight week-old female C57BL/6J (CD45.2+), congenic C57BL/6J (B6.SJL-PtprcaPep3b/BoyJ) (B6.Ly5.1) (CD45.1+), Aicda-ERT2-cre (B6.129P2-Aicdatm1.1(cre/ERT2)Crey/J), Rosa26-stop-tdTomato (B6.Cg-Gt(ROSA)26Sortm14(CAG-tdTomato)Hze/J), Prdm1-EYFP (B6.Cg-Tg(Prdm1-EYFP)1Mnz/J), K18-hACE2 (B6.Cg-Tg(K18-ACE2)2Prlmn/J) mice were purchased from the Jackson Laboratory and S1pr2-ERT2-cre mice generated by T. Kurosaki and T. Oakda were kindly provided by V. Gabriel. Six to eight week-old male C57BL6/J mice were used for sex difference experiments (Extended Data Fig. 2e-g). All mice were housed on the 12-hour light cycle at 72 degrees Fahrenheit and at 50% humidity.
Wild animals	No wild animals were used in this study.
Reporting on sex	No sex differences were found in this study.
Field-collected samples	No field-collected samples were used in this study.
Ethics oversight	All procedures used in this study complied with federal guidelines and institutional policies by the Yale animal care and use committee. All procedures for propagation and infection of SARS-CoV-2 were performed in a BSL-3 facility with approval from the Yale Institutional Animal Care and Use Committee and Yale Environmental Health and Safety.

Note that full information on the approval of the study protocol must also be provided in the manuscript.

Plants

Seed stocks: n/a

Novel plant genotypes: n/a

Authentication: n/a

Flow Cytometry

Plots

Confirm that:

☒ The axis labels state the marker and fluorochrome used (e.g. CD4-FITC).

☒ The axis scales are clearly visible. Include numbers along axes only for bottom left plot of group (a 'group' is an analysis of identical markers).

☒ All plots are contour plots with outliers or pseudocolor plots.

☒ A numerical value for number of cells or percentage (with statistics) is provided.

Methodology

Sample preparation: Lungs were minced with scissors and incubated in a digestion cocktail containing collagenase A (1 mg/mL, Roche) and DNase I (30 ug/mL, Sigma-Aldrich) in PBS at 37°C for 45 min under gentle agitation followed by filter with 70-um mesh. After centrifugation at 600 g for 3 min at 4°C, pellets were treated with ammonium-chloride-potassium (ACK) buffer to lyse red blood cells (RBCs) at room temperature for 2 min and washed with FACS buffer (PBS with 3% FBS). Following centrifugation, pellets were resuspended with FACS buffer for downstream analysis.

Instrument: LSR II flow cytometer (BD)

Software: BD FACSDiva Software v9.3.1, FlowJo v10

Cell population abundance: For scRNA-seq analysi, IV negative and CD45 positive cells were sorted.
For qPCR analysis, CD19+ cells were purified using EasySep Mouse B cell Isolation Kit (STEMCELL Technologies) with more than 90% purity.

Gating strategy: For all experiments, we identified cells by gating on lymphocytes using FSC-A and SSC-A. Doublets were excluded using SSC-A and SSC-H. For extravascular immune cell analysis, i.v. CD45+ cells were gated out. All immune cells were gated using specific cell surface and intracellular markers. We defined positive and negative population at the clear distinct border. Gating strategies for antigen-specific T and B cells in the lung were included in Extended Data Fig 1.

☒ Tick this box to confirm that a figure exemplifying the gating strategy is provided in the Supplementary Information.
